# The Peptonizer2000: Bringing Confidence to Metaproteomics

**DOI:** 10.1021/acs.jproteome.5c00567

**Published:** 2026-03-04

**Authors:** Tanja Holstein, Pieter Verschaffelt, Tim Van Den Bossche, Simon Van de Vyver, Lennart Martens, Bart Mesuere, Thilo Muth

**Affiliations:** † 26656 VIB-UGent Center for Medical Biotechnology, VIB, Zwijnaarde 9052, Belgium; ‡ Department of Biomolecular Medicine, Ghent University, Ghent B-9000, Belgium; § Data Competence Center MF 2, Robert Koch Institute, Berlin 13353, Germany; ∥ BioOrganic Mass Spectrometry Laboratory (LSMBO), IPHC UMR 7178, University of Strasbourg, CNRS, Strasbourg 67000, France; ⊥ Infrastructure Nationale de Protéomique ProFI - FR2048, Strasbourg 67087, France; # Department of Applied Mathematics, Computer Science and Statistics, Ghent University, Ghent 9000, Belgium

**Keywords:** metaproteomics, graphical model, taxonomic inference, microbiome analysis

## Abstract

Metaproteomics, the large-scale study of proteins from microbial communities, faces challenges in identifying species due to similarities in protein sequences across different organisms. Current methods often rely on simple counting of matches between proteins and taxa, which can lead to low accuracy. We introduce the Peptonizer2000, a new tool that uses advanced modeling to provide more precise taxonomic identifications along with confidence scores. It combines peptide scores from any proteomic search engine with peptide-to-taxon links from the Unipept database. By applying statistical models, the Peptonizer2000 improves taxonomic resolution and delivers more reliable results. We validate its performance using publicly available data sets, demonstrating its ability to produce high-confidence identifications. Our results suggest that the Peptonizer2000 improves the specificity and confidence of taxonomic assignments in metaproteomics, providing a valuable resource for the study of complex microbial communities.

## Graphical abstract

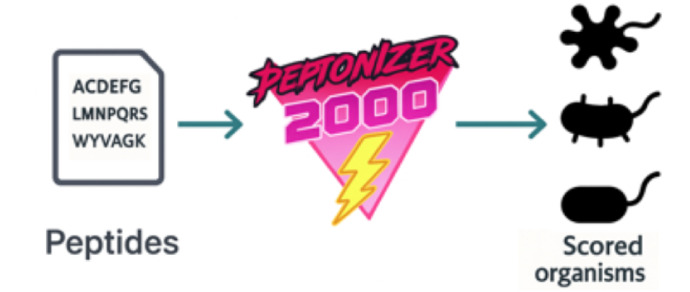

## Introduction

Microbial communities, also known as microbiomes, can be found in diverse environments such as ocean water,^1^ biogas plants,^2^ and the human gut.^3^ Recent technological and bioinformatic advances have enabled new discoveries such as the ability of microbiomes to use carbon monoxide in certain marine worms,^4^ insights into the composition of certain environmental microbiomes,^5^ the alteration of the gut microbiome depending on diet,^6^ and, more generally, the role microbiomes play in health and disease of humans^7^ and animals.^8^ Metaproteomics is a growing field that studies microbiomes by directly analyzing their proteins to investigate microbiome function and taxonomic composition.^9–11^ Tandem mass spectrometry (MS/MS), a technology from traditional proteomics, is employed to analyze proteins in microbiome samples. However, microbiome samples are more complex to handle and understand than single-organism samples, which poses challenges in experimental and bioinformatic setups.^12,13^

One key objective of metaproteomics is to identify the microorganisms present in the sample along with their roles. This task is complicated by the fact that proteins often share similarities in their sequences and that they can be linked to multiple groups of organisms.^14^ In very complex samples, identifying specific species or even families of microorganisms can be challenging.

Several bioinformatic tools have been developed to identify taxa and functions in microbiome samples. Among the most popular software tools are MetaProteomeAnalyzer,^15^ iMetaLab,^16^ the Galaxy framework,^17^ and Unipept.^18^ Notably, some of these workflows are peptide-centric, e.g., Unipept, tallying peptide–taxon matches without explicit protein inference, whereas protein-centric alternatives, e.g., iMetalab, first infer protein (group) presence and then assign taxa. Each paradigm has trade-offs: peptide-centric methods minimize inference complexity but face ambiguity from shared peptides, while protein-centric methods may gain specificity at the expense of additional assumptions and potential bias from grouping strategies.^19^ Additionally, the large comparative CAMPI study concluded that, because in complex samples many proteins are very similar across species, peptides are often shared across taxa; inferring taxa directly from peptides tends to produce more stringent taxonomy filtering.^20^ Returning to practice, many workflows–regardless of centricity–ultimately adopt simple presence rules, most commonly declaring a taxon present once a minimum number of observations is present (typically two or three per taxon).^21–23^ These methods disregard underlying uncertainties inherently linked to any data collection method and may suggest a false sense of confidence in the obtained results. Additionally, when a peptide cannot be assigned to a specific species, it is mapped to the lowest taxonomic level to which it is specific, reducing the taxonomic resolution of the analysis. This taxonomic level is referred to as the lowest common ancestor (LCA).^24^ The LCA approach is key to the taxonomic identification performed by Unipept. Over recent years, the number of taxa and associated proteins in publicly available databases like UniProt has steadily increased from 51 million in 2014 to 245 million in 2024.^25^ This growth has been shown to decrease the specificity of peptides at lower taxonomic levels, thus reducing the taxonomic resolution of the LCA approach.^18^

Addressing these issues requires more sophisticated taxonomic identification algorithms. MiCiD^26^ is one such method, providing integrated metaproteome analysis supported by rigorous statistical methods for accurate taxonomic identification. However, MiCiD uses its own built-in database search for mass spectrum identification, which is tightly linked to its taxonomic assignment process. As a result, it is not compatible with outputs from other widely used search engines like X!Tandem^27^ or rescoring tools like MS2Rescore^28^ and is restricted to data-dependent acquisition (DDA) spectra only.

In contrast, Unipept can flexibly process peptide lists from any search engine but does not incorporate peptide scores in its analysis. Instead, it relies on counting peptides assigned to the LCA for taxonomic inference, which limits its precision.

None of the aforementioned software solutions combine advanced statistics, straightforward usability, comprehensive visualization options, and compatibility with any preferred search engine. Furthermore, they lack support for both DDA and data-independent acquisition (DIA) data despite DIA’s increasing popularity in recent metaproteomics studies.^21,29,30^

We introduce the Peptonizer2000, a novel workflow for taxonomic identification of metaproteome samples. Unlike previous approaches, the Peptonizer2000 models the joint probability distribution of all detected peptides and the potentially present taxa using a graph representation. As an alternative to arbitrary heuristics, it uses a Bayesian approach to compute the presence of taxa by calculating their marginal probabilities. This results in taxonomic identifications with associated confidence scores. Conceptually, the Peptonizer2000 is peptide-centric: it models peptide evidence directly rather than committing to a prior protein-inference step. This choice preserves sensitivity in complex communities where extensive peptide sharing makes protein grouping unstable, while our Bayesian framework compensates by propagating peptide-level uncertainty to calibrated taxon-level probabilities. We previously developed PepGM,^31^ a graphical model-based workflow for the taxonomic identification of single-virus proteome samples. In that work, we addressed the challenge of strong protein sequence homology between viral strains, which can make accurate strain-level identification difficult, an issue that is analogous to the one faced here when identifying the species present in microbiomes. In this study, we have shown that using a more advanced statistical model leads to more accurate taxonomic assignments. Building on this, the Peptonizer2000 integrates statistical modeling for taxonomic assignments, incorporating peptide scores from proteomic search engines along with peptide-taxon mapping from Unipept. This allows the Peptonizer2000 to provide high-resolution taxonomic identification for microbiome samples, including probability scores for each taxon. By doing so, the Peptonizer2000 adds confidence to taxonomic assignments that were, until now, reliant on discretionary PSM cutoffs.

We show that our new approach can compute taxonomic confidence scores at user-selected taxonomic levels for various metaproteomics samples using different background databases. Comparing the Peptonizer2000’s probabilistic identifications with Unipept’s PSM-based counts, we demonstrate how these confidence scores offer valuable evidence to distinguish between truly present taxa and spurious taxon-peptide matches.

## Methods

### The Peptonizer2000 Workflow

A broad overview of the Peptonizer2000 workflow is shown in Figure 1, which consists of three main steps: first, querying all candidate peptides in the Unipept database and collecting candidate taxa based on weighted PSMs; second, constructing the graphical model, performing belief propagation, and conducting a grid search through graphical model parameters; and third, evaluating the parameters, followed by output and visualization of the results. These steps are adapted from our previously developed taxonomic identification tool, PepGM.

**Figure 1:**
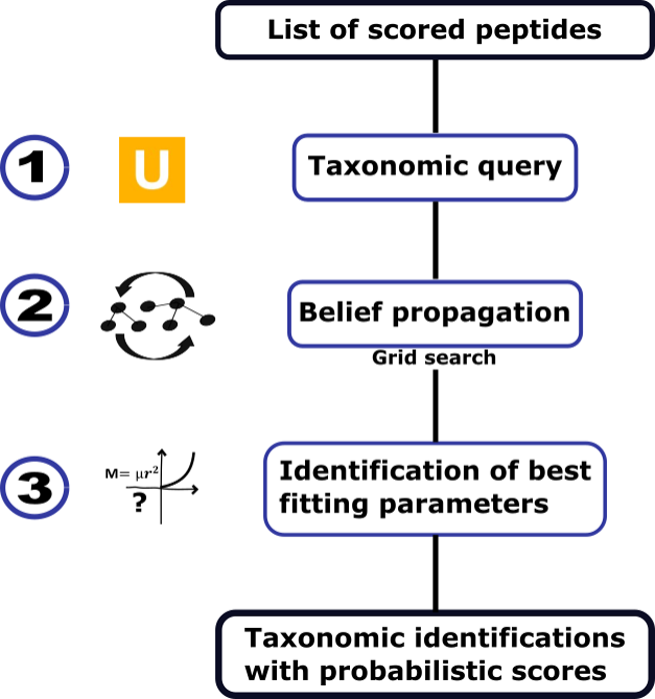
Overview of the Peptonizer2000 workflow. A list of scored peptides has to be provided as input. (1) These peptides are queried for taxonomic information using Unipept. (2) Using a factor graph, the probabilities at the peptide level are propagated to the taxa. Model parameters are evaluated through a grid search. (3) The optimal parameters for each sample are determined using a custom metric, resulting in taxonomic identifications with associated confidence scores.

Snakemake^32^ is used as the workflow management system, while all scripts are written in Python and developed and tested for the Linux operating system and Python version 3.10. All additional Python packages used are listed in the Supporting Information.

The Peptonizer2000 is available at https://github.com/compomics/Peptonizer2000. The code used to generate the tree view graphics of the results can be found at https://github.com/pverscha/peptonizer-visualizations.

We provide a detailed description of the workflow and algorithms used below.

### Required Input

The Peptonizer2000 is a comprehensive workflow designed to analyze metaproteomics data. The workflow starts with an input file provided by the user, which contains the results of a metaproteomics sample analysis. This input file uses a generic tabular format, with one column listing peptides and a second column providing a corresponding score for each peptide. These scores represent the probability of each peptide to truly be present in the sample, as, for instance, reported by the search engine. This means that, currently, neither peptide abundances nor the specific number of spectrum matches per peptide are taken into account—the Peptonizer2000 relies on sequences and confidence scores only. If the same peptide appears multiple times with different scores in the input list, the highest score is kept for downstream analysis. Note that this flexible input format allows the user to apply the Peptonizer2000 workflow to search results from both DDA and DIA methodologies. Additionally, even peptides resulting from *de novo* sequencing are supported.

### (1) Unipept Query for Taxonomic Annotation of Peptides

The workflow starts by querying the Unipept database to retrieve taxonomic annotations for each peptide. All peptides provided are submitted to Unipept through a newly developed API end point. This API returns all taxa associated with a peptide based on its UniProt^25^ annotation without computing the LCA taxon, as was previously the case. The end point provides a list of all species and strains whose proteomes contain the queried peptide.

Peptides mapping to more than 10,000 proteins are excluded from the search. Peptides associated with that many proteins are likely part of so-called housekeeping proteins,^33^ which contribute minimal taxonomic information. A preliminary investigation into the LCA of the excluded proteins is shown in the Supporting Information, specifically in the section”Taxonomic rank of peptides and proteins excluded by the Peptonizer” and confirms this observation.

The length of the peptides queried also changes taxonomic resolution, which is intrinsically taken into account by the structure of the downstream graphical model. We include a brief exploration of how peptide length affects taxonomic specificity in the Supporting Information (Figures S1–S2). Notably, the taxonomic range of the query can be refined by specifying a set of species, enabling users to limit the search based on prior knowledge about potentially present microorganisms in the sample. Users can restrict the taxa of interest at any taxonomic level by specifying their corresponding taxid (NCBI unique taxon identifier). For example, if the specified taxid is 2, the Unipept query will be limited to bacterial reference proteomes. In all our analyses, we did not restrict the taxonomy of the Unipept query. This approach simulates a scenario where researchers lack prior knowledge about the microbial composition of the sample.

The querying process results in a response from Unipept in JSON format, containing taxonomic information linked to the peptides, their scores, and the number of PSMs. This output is saved as an intermediate file and serves as input for the following step.

#### Selection of Candidate Taxa

Peptides from a metaproteomic sample can map to a wide range of taxa, influenced by both sample complexity and the taxonomic range selected for querying. An unrestricted taxonomic query often results in a graph with over 1,000 potentially present organisms, leading to highly increased computation times. The number of potentially present organisms is so high because, commonly, certain peptides from protein sequence regions that are highly conserved across species are frequently detected alongside more species-specific peptides.

To handle this complexity, we restrict the number of taxa included in the graph. By default, this number is set to 150; users can customize this number through the configuration file. Every taxon with at least one unique peptide will be included in the graph, even if this results in exceeding the default limit of 150 taxa. The 150 taxa to be included are selected from the candidate taxa attributed the highest weight, which is is calculated as detailed in the following.

The number of peptides detected for a present taxon exhibits a large dynamic range. It depends both on the organism’s size and the relative abundance of the organism within the sample. For instance, in an MS run involving the commonly used, lab-assembled SIHUMIx mixture,^34^ 5 PSMs were detected for *Lactobacillus plantarum*, while over 1,000 PSMs mapped to the more abundant *Bacteroides thetaiotaomicron*. Both taxa are present in the SIHUMIx mixture. We aim to address this issue by assigning a high weight to unique PSMs. This approach makes sure that both the number of spectra matching to a peptide and the peptide’s taxonomic information, including its sequence degeneracy, are considered.

The formula to compute the weight *W*_T_ of a taxon is the following:

1 $$W_{T}=\underset{i}{\sum }\frac{\#{\mathrm{PSM}}_{i}}{Deg_{i}}$$

where *i* stands for all peptides mapped to taxon T, #PSM_*i*_ denotes the number of PSMs for peptide *i*, and *Deg*_*i*_ is the degeneracy of peptide *i*—specifically, the number of taxa to which the peptide has been mapped.

All candidate taxa and their corresponding scored peptides are exported as a CSV file, which serves as the basis for the graphical model.

### (2) Assembly of the Graphical Model, Belief Propagation, and Grid Search

#### Graphical Model Assembly

The construction of the graphical model, which is a factor graph, uses the same algorithm as PepGM. All peptides and taxa from step (2) are included in the graph. Two categories of nodes represent peptides and taxa, respectively. An edge is drawn between a peptide node and a taxon node if a peptide is part of a taxon’s proteome. As described in Holstein et al.,^31^ factor nodes based on the noisy-OR model and convolution tree nodes are added to the graph. Together, the graphical model represents the probability distribution of all peptides and taxa. Note that this probability distribution is an approximation, as not all taxa are included in the graph, and it relies on the same assumptions regarding peptide emission and detection probabilities as in PepGM. The noisy-OR model introduces three parameters:

- α: The probability of observing a peptide given the presence of its parent taxon.
- β: The probability of a peptide being randomly or falsely observed or incorrectly linked to a taxon that is not actually present in the sample.
- γ: The prior probability of a taxon being present.

We have discussed the choice and use of these parameters more extensively for PepGM.^31^

In the Peptonizer2000, we introduce an additional option to regularize the probabilities for a peptide having *n* parent taxa present in the noisy-OR model. This regularization means that the probabilities for a peptide to have *N* parent taxa present are assumed to decrease inversely proportional to *N*. This assumption has been previously applied for protein inference^35^ models probability distribution for samples where a very broad taxonomic range was queried compared to the expected number of present taxa. The user can choose to turn this regularization on or off. In metaproteomics, reference databases typically contain far more taxa than are actually present or detected in the sample. For this reason, we generally recommend keeping the regularization option activated for smaller lab-assembled mixtures. For complex biological samples, we recommend to keep the regularization option off.

#### Inference Algorithm and Grid Search

To compute the marginal probabilities of taxa, we use the belief propagation algorithm.^36^ To efficiently handle the large size of the graph, we implemented a version of belief propagation known as “zero-lookahead”.^37^ This version of belief propagation was shown to scale nearly linearly with graph size while introducing only a slight approximation of the global probability distribution.

Additionally, we perform a grid search across the three model parameters introduced by the noisy-OR model. To reduce the search space, the prior probability is set to the following values: γ ∈ [0.1, 0.3, 0.5]. The peptide emission probability is set to α ∈ [0.85, 0.9, 0.99]. The error probability is set to β ∈ [0.5, 0.6, 0.7] by default. However, these values can be modified by the user. The rationale behind the selection of these parameters, as well as their interpretation, is discussed in the Results section. The optimal parameters for a specific sample are determined in the following step.

### (3) Selection of Most Appropriate Parameter Range

For each combination of model parameters α, β, and γ, marginal probabilities are computed for all taxa in the graphical model. To identify the optimal set of parameters for the given sample, a thorough evaluation of these parameters is conducted.

The evaluation of the best fitting parameters is based on a comparison between a predefined list of weighted taxa used as input for the graphical model and the results of the belief propagation from Peptonizer2000. In the following, we refer to the weighted list as *L*_W_ and the scored list as *L*_S_. Both lists of taxa are ranked according to their weights and scores, respectively.

Since the weights assigned to each taxon rely on the number and uniqueness of PSMs per taxon, the weighted taxon list *L*_W_ gives an initial indication of the taxa likely to be present. However, it has one major limitation: closely related taxa, such as multiple highly similar bacterial species, may all appear near the top of the list in *L*_W_. To resolve this, we aim to select a parameter range that can distinguish between these taxa.

The solution we propose is to cluster *L*_W_ based on taxon similarity. The clustering procedure is analogous to the taxonomic clustering method previously described in Alves and Yu,^26^ with one key modification: instead of relying on the full theoretical peptidome, the similarity between two taxa is based solely on their detected peptides. This adjustment ensures that the clustering is closer to the currently analyzed sample, where taxon abundances and the number of detected peptides may vary greatly.

The taxon with the highest number of detected peptides within a group of similar taxa is designated as the cluster head. A threshold similarity of 0.9 is used to group taxa into clusters—this threshold is arbitrary but was chosen to ensure that closely related taxa are clustered together. While the threshold should be well above 0.5 for meaningful clustering, a perfect similarity score of 1 is not required. In fact, slight variations in the threshold of up to 0.1 result in changes of only one or two taxa in the clustered taxon list. After taxon clustering, we obtain a simplified version of the weighted taxon list *L*_W_, which contains only the weight-sorted cluster heads.

Both lists are then ready to be compared. For the comparison, we need to take into account the following: taxa listed in the results of Peptonizer2000 *L*_S_ may be absent from the clustered list *L*_W_. Thus, we need a comparison method that allows for nonconjointness. Additionally, higher-ranked taxa in both lists are more important than lower-ranked ones, meaning that taxa with higher weights and scores should have a greater influence on the comparison. To meet these criteria, we use rank-biased overlap^38^ (denoted RBO) as the similarity measure for list comparison. It handles nonconjointness and gives more weight to the top-ranked taxa in both lists.

The optimal parameter range for a given sample is selected by maximizing the rank-biased overlap between the clustered *L*_W_ and the scored results *L*_S_ while simultaneously minimizing the entropy of the marginal probability distribution. Entropy, denoted as *S*, has been previously used as a criterion in PepGM,^31^ and its inclusion here aims to maximize the information content of the resulting distribution.

Thus, the final metric that the chosen parameter range for each sample should maximize is given by

2 $$M=RBO\cdot S^{-2}$$

By comparing *M* across different parameter ranges, the one with the highest value can be selected, ensuring both strong alignment between *L*_W_ and *L*_S_ and a low-entropy, high-information distribution.

### Result Outputs and Visualization

Peptonizer2000 outputs results in various formats. The most basic output is a CSV file containing a list of all identified taxa with their corresponding score, which is the most basic output level. A bar chart summarizes the results as a .png, alongside a second bar chart that shows the performance of each parameter set based on the rank-biased overlap and entropy metric.

A visually appealing tree view of the results can also be generated in both HTML and SVG formats, offering an interactive and high-quality graphical representation of the data.

## Materials

We evaluated Peptonizer2000 using several publicly available data sets from metaproteomic samples, which can be accessed through the PRIDE database.^39^ The first set of samples was obtained from the CAMPI study,^20^ all available under the PRIDE identifier PXD023217.

The first sample type we analyze is the SIHUMIx sample.^40^ It was developed as a model community for the human gut microbiota and covers the three dominant genera found in human feces: Firmicutes, Proteobacteria, and Bacteroidetes. It contains eight microbes: *Bacteroides thetaiotaomicron*, *Anaerostipes caccae*, *Escherischia coli*, *Lactoplantibacillus plantarium*, *Clostridium butyricum*, *Thomasclavelia ramosa*, *Blautia producta*, and *Bifidobacterium longum*. SIHUMIx is widely used for benchmarking in metaproteomics. Analogous to the original CAMPI publication, we use samples S03, S05, S07, S08, and S11 for our investigation, referring to them collectively as S03–S11. Each sample was acquired using different laboratory workflows, resulting in slightly varying coverage of the proteomes contained in the samples.

The second sample type taken from the CAMPI study is a fecal sample, for which the taxonomic “ground truth” is unknown. For our analysis, we chose the fecal sample denoted as F07.

Another set of samples we analyze consists of a more complex, lab-assembled mixture of up to 32 different microorganisms.^41^ These samples are available on PRIDE under the identifier PXD006118. For our analysis, we selected samples from three types of communities: those assembled with equal cell amounts per taxon (denoted “C1”), equal protein amounts per taxon (denoted “P1”), and uneven composition (denoted “U1”).

To include samples from diverse environments, we also analyzed one ocean microbiome^42^ under PRIDE identifier PXD043218 and one soil microbiome^43^ at the identifier PXD026798. Respectively, we randomly selected the samples *Lab_127_1.mgf* and *Q08425_Bouafles18–3_8a.mgf* for our analyses. We used the databases provided through the respective PRIDE repositories.

For the rest of our evaluation, we used four different reference databases. The first is the SIHUMIx database, specifically tailored to the SIHUMIx sample, which is accessible through the corresponding PRIDE repository associated with the CAMPI study. The second database is the integrated gene catalog (IGC),^44^ a gut microbiota reference database that includes representative sequences aiming to cover the majority of gut microbes. The third database is tailored to the 32 potentially present microorganisms in the complex mock community and was provided alongside the experimental data of the Kleiner et al. study.^41^ These three databases are concatenated with a contaminants database^45^ to ensure that peptide hits matching the contaminant sequences will be filtered out before taxonomic analysis.

Finally, as a fourth reference, we use the UniRef90 database,^46^ a clustered set of sequences derived from UniProt, aiming to maintain comprehensive coverage of the sequence space while reducing redundancy.

We analyzed all samples beginning with a standard database search followed by rescoring. The MS/MS spectra are searched against the provided reference database using a combination of Sage^27^ and MS2Rescore.^28^ This process generates a list of identified peptide-spectrum matches along with their corresponding scores. MS2Rescore is employed to enhance the reliability of these identifications. It rescales the initial scores based on statistical models and provides e-values that represent the probability of false detection for each peptide.

For the proteomic database search using Sage, we fixed the search parameters in accordance with the original sample publications. The parameters were the same for all samples analyzed and included specific trypsin digestion with a maximum of two missed cleavages, mass tolerances of 10.0 ppm for MS1 and 0.02 Da for MS2, and fixed modifications of carbamidomethylation of cysteine (+57.021464 Da). Variable modifications included oxidation of methionine (+15.994915 Da). Additionally, we set the peptide length to range between 6 and 50 amino acids and included charge states of +2, + 3, or +4. A false discovery rate (FDR) threshold of 1% was also applied to ensure the accuracy of the identified peptides.

To perform the Unipept analysis, we used the Unipept command-line tool with the option “–pept2lca”, taking a .txt file containing all peptides that were provided as input for Peptonizer2000. Since Unipept outputs the LCA for the queried peptides, we filtered the Unipept response to retain only those peptides that are unique to taxa at the species level. We then aggregated the number of unique peptides per species to obtain taxonomic identifications at the species level based on unique PSMs. For analyses at higher taxonomic levels, we used the same approach, mapping unique peptides at lower taxonomic levels back to their corresponding ancestors.

## Results

### Taxonomic Identification Results for Samples with Known Composition and Varying Complexity Showcase Reliable Confidence Estimates

To highlight the benefits of probabilistic scoring for taxonomic identification and to demonstrate the accuracy of the Peptonizer2000, we analyzed a selection of publicly available data sets using both Unipept and the Peptonizer2000.

#### Taxonomic Composition of Lab-Assembled Mixtures

For initial testing, we used lab-assembled mixtures with a defined taxonomic composition, providing a clear ground truth for the species present in the samples. This means that we can clearly assess the Peptonizer2000’s performance: if all taxa known to be in the ground truth are correctly reported, and only those, the Peptonizer2000 performs well. We selected two publicly available data sets: one (referred to as the SIHUMIx sample) from the CAMPI study,^20^ a large, cross-laboratory metaproteomic study from the Metaproteomics Initiative,^47^ and the other from a study that assesses methods for estimating biomass contributions in microbiomes.^41^ We began our analysis with the provided spectral and database files from the respective data sets, performing the database search and rescoring as detailed in the Materials section.

#### Taxonomic Composition Results for the SIHUMIx Sample

We analyzed the taxonomic composition of the samples S03, S05, S07, S08, and S11 from the SIHUMIx data set of the CAMPI study. SIHUMIx is composed of *Bacteroides thetaiotaomicron*, *Anaerostipes caccae*, *Escherischia coli*, *Lactoplantibacillus plantarium*, *Clostridium butyricum*, *Thomasclavelia ramosa*, *Blautia producta*, and *Bifidobacterium longum*. As a reference database, we used a sample-specific SIHUMIx database, provided alongside the MS/MS data in the original study. This database includes *Blautia pseudococcoides* as the representative species of the genus *Blautia*, as this is the available reference proteome. In the following, we therefore also accept *Blautia pseudococcoides* as a valid taxonomic identification result.

Figure 2 shows the analysis results for the top-scoring taxa in samples S07 (A) and S11 (B). The Peptonizer2000 correctly identifies all present taxa as top-scoring, with probability scores ranging from *p* = 1 to *p* = 0.9. Additionally, all present taxa are accurately represented among those with the highest number of unique peptides. The results also demonstrate several notable attributes of both Peptonizer2000 and Unipept, which we will explain in the following.

**Figure 2:**
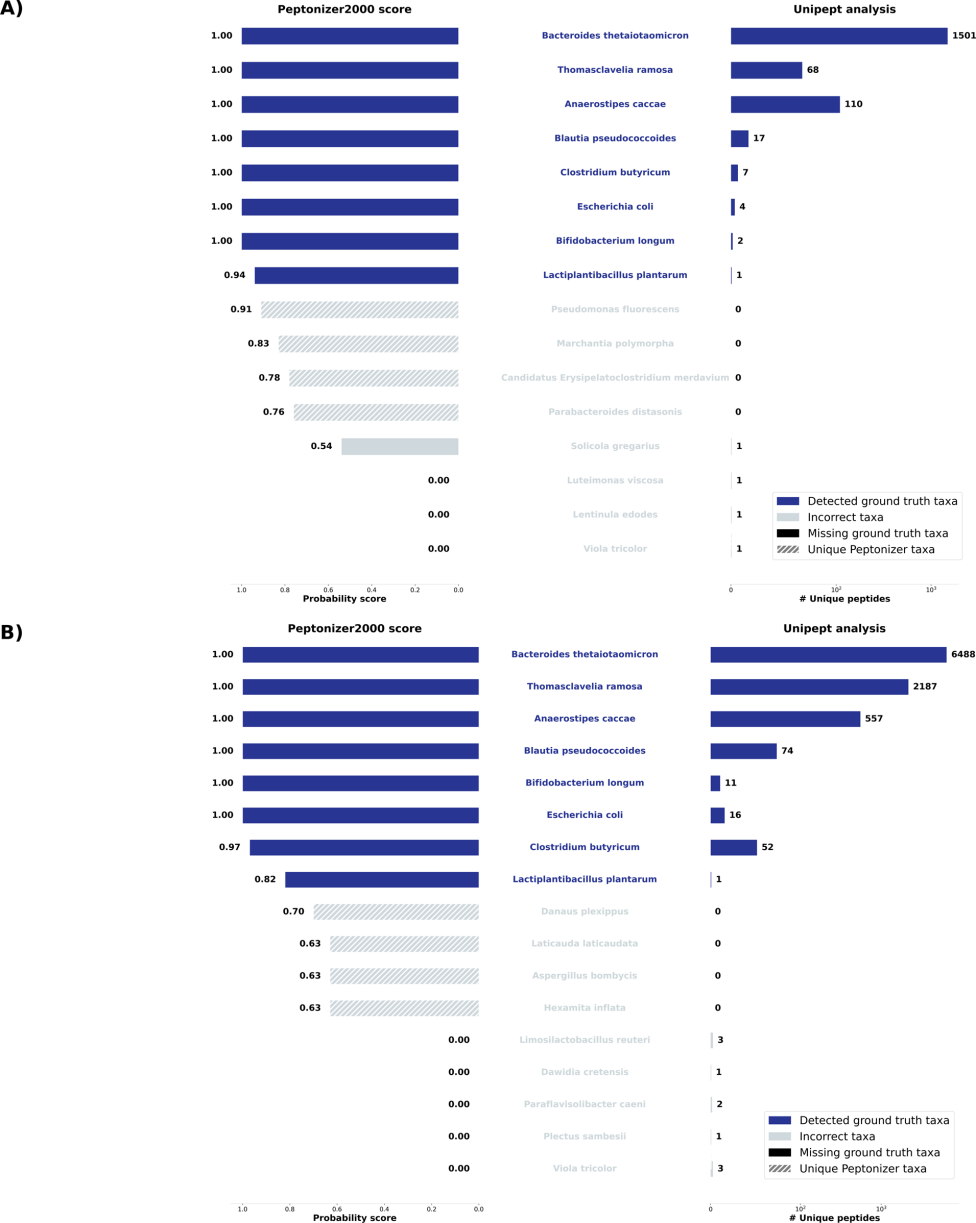
Taxonomic identification results for the SIHUMIx samples (A) S07 and (B) S11. The left panels display the results from Peptonizer2000, including corresponding probability scores, while the right panels show results based on unique peptides from Unipept. Dark blue shades indicate correct taxonomic identifications. The two software tools demonstrate a high degree of overlap in their results.

The Unipept results show a very high range in the number of detected peptides (between 1 and 6488) for the taxa present in the sample. From the Unipept results alone, selecting the present taxa with arbitrary criteria like “a minimum of 2 unique peptides per taxon” is hard to justify, even though in the present case, it would lead to correct identifications. This type of cutoff is very common in metaproteomic data analysis.^21–23,48,49^

The Peptonizer2000 assigns all the highest scores to the correct taxa. Since they represent a probability distribution, introducing a “cutoff” probability for present taxa does not make sense mathematically but will often be necessary for practical reasons. We analyzed precision, recall, and F1 score which are shown in Figures S6 and S7 in the Supporting Information. The F1 score suggests a cutoff of 0.8 for sample S11 and 0.9 for sample S07. The highest F1 score for both combined can be achieved through the combination of both results, with the criterion “Peptonizer score of *s* > 0.8 and at least one unique peptide detected”, as shown in Figure 3 and Supplementary Figure S8.

**Figure 3:**
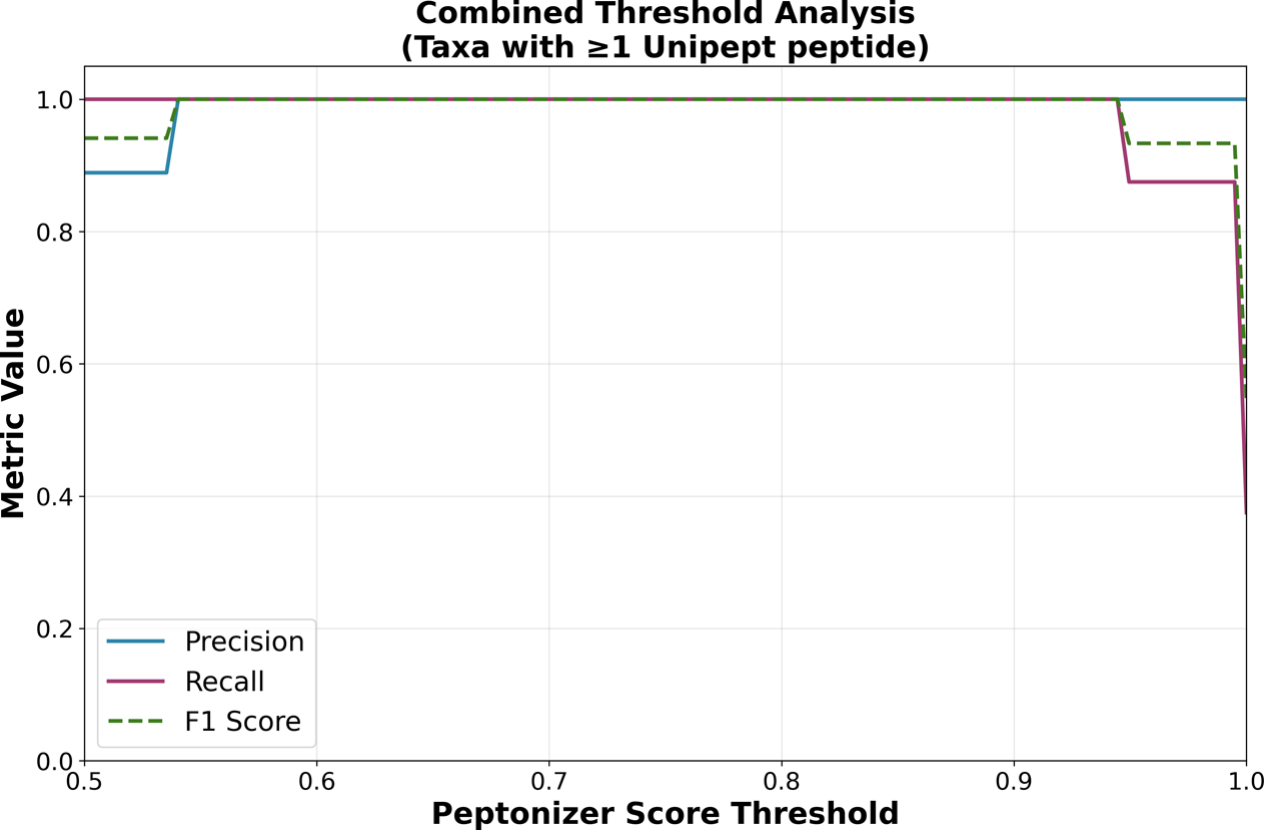
Precision, recall, and F1 score depending on different Peptonizer score thresholds, for taxa with at least one unique peptide, shown for sample S07.

Similar conclusions can be drawn from the Peptonizer2000 and Unipept results for the samples S03, S05, and S08 (Supplementary Figures S3–S4).

#### Taxonomic Composition Results for More Complex Samples

To assess the performance of the Peptonizer2000 on complex samples, we analyzed a data set from a previously studied lab-assembled mixture containing up to 32 microorganisms, as reported in a prior metaproteomic study.^41^ We examined samples U1, C1, and P1, which were constructed with uneven taxonomic abundance, protein, and cell number (U1), equal cell amount (C1), and equal protein amount (P1).

Figure 4A shows the Peptonizer2000 identification results for sample U1 in a treeview diagram, displaying all taxa with a probability score above 0.8. Additionally, Figure 4B illustrates the Peptonizer2000-attributed scores for all taxa scoring above 0.8 together with the 30 taxa with the highest number of unique peptides using Unipept.

**Figure 4:**
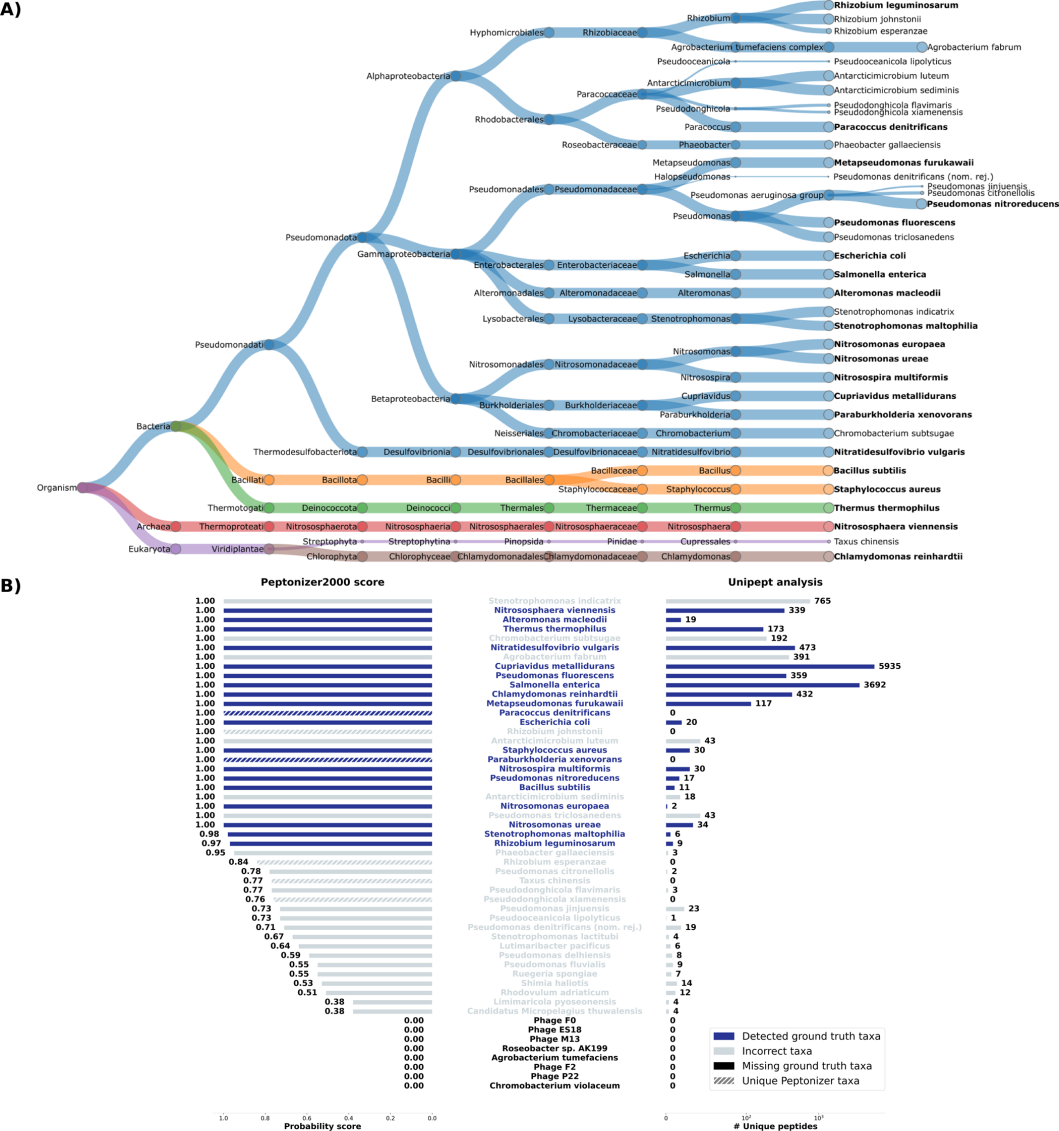
(A) Taxonomic tree view of the Peptonizer2000 results for a lab-assembled sample of 28 different species, filtered with a probability score above 0.8. Node sizes are proportional to probabilities attributed by the Peptonizer2000. Correct taxonomic identifications are displayed in bold font. (B) Comparison of the Peptonizer2000 probability scores (left) and the number of unique peptides detected per taxon using Unipept (right). Correct identifications are shaded in darker blue.

Of the 32 potentially present taxa, 28 were included in the lab-assembled mixture by the original study designers. Among these, 5 are phages, which we did not detect, likely due to their very low protein amounts and the filtering steps currently applied by Unipept. Additionally, taxonomic annotations for viruses and phages in reference databases are often less comprehensive and less curated than those for bacteria and eukaryotes.^50^ Of the remaining 23 microorganisms, 20 were correctly identified. The three missing taxa are *Roseobacter* *sp. 199*, *Agrobacterium tumefaciens*, and *Chromobacterium violaceum*. The species *Roseobacter* *sp. 199* is currently absent in the NCBI taxonomy, which could explain its absence from the results. *Agrobacterium fabrum* is a heterotypic synonym of *Agrobacterium tumefaciens* and could therefore be evaluated as a correct identification. Some erroneous taxa received scores above 0.8. Most of these are closely related to the present species. The Peptonizer2000 cannot distinguish between certain very closely related species on the detected peptides. Notably, *Stenotrophomonas indicatrix* has 561 unique peptides attributed to it by Unipept, even though it is not present in the sample. Because it is unlikely that there are 561 incorrect PSMs, this is likely due to either misannotations present in the UniProt database or a wrongly annotated strain in the reference database we used for the database search. Again, we analyzed precision, recall, and F1 score, shown in the Supporting Information in Figure S10. The optimal Peptonizer score seems to be at around *s* = 0.85. Contrary to less complex samples, we cannot recommend setting a minimum of one unique peptide to identify taxa.

The comparison between the Peptonizer2000 and Unipept results in Figure 4 highlights the advantage of integrating their outputs. The Peptonizer2000 scores provide valuable insights into the presence or absence of taxa with unique peptides, especially considering the broad range of unique peptides mapped to identified taxa (from 2 to 5000). In the current sample, the Peptonizer2000 scores confirm the presence of *Paracoccus denitrificans* and the absence of *Pseudomonas nitrireducens*, even though both taxa have six unique peptides attributed to them. However, some false positives persist. For example, *Antarctimicrobium sediminis* obtains a high probability score of 0.96 and has 18 unique peptides despite not being present in the sample. Additionally, two archaeal species are identified as false positives in the Peptonizer2000 results, one of which includes *Candidatus* in its name, suggesting potential reference database issues. Since archaea are frequently misidentified, implementing an optional filter for “archaea” taxa could be a useful feature in future updates.

The taxonomic analysis of samples C1 and P1 is presented in the Supporting Information (Figures 4 and 5). While the number of correct identifications is lower—18 for community C1 and 16 for community P1—the findings regarding the complementary value of unique PSMs from Unipept and Peptonizer2000 scores still hold true. The effect of including shared peptides on taxonomic assignment is evident. Consider, for example, *Paraburkholderia xenovorans*: it receives a score of 1 but has no unique peptides according to Unipept. A closer look at the peptide-taxon mappings internal to the Peptonizer2000 can explain this. The Unipept command-line tool used in this analysis outputs the LCA for each queried peptide. Consider the peptide “QAGQMR”. It maps to *Paraburkholderia xenovorans*, and by chance to another very distant taxon *Y*, it is LCA is “root”. The Peptonizer, on the other hand, includes taxa into it is graph based on peptide-count-related weight. *Paraburkholderia xenovorans* attained a weight above an internal threshold, while taxon *Y* did not. This results in the peptide “QAGQMR” uniquely mapping to *Paraburkholderia xenovorans* in the Peptonizer’s internal graph. Additionally, *Paraburkholderia xenovorans* has many shared peptide evidence supporting its presence, which leads to the high score the Peptonizer’s inference algorithm attributes it.

**Figure 5:**
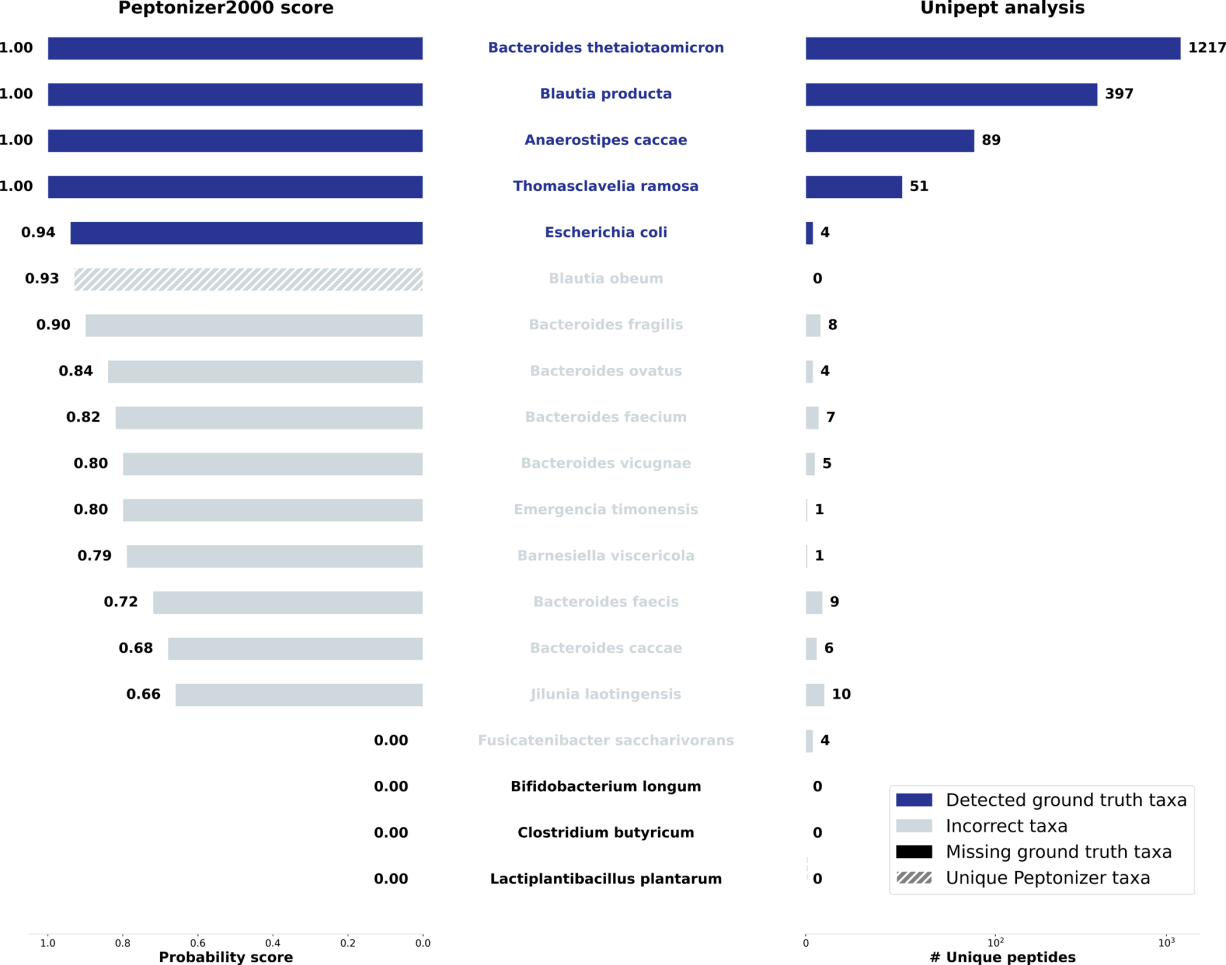
Taxonomic identification of the SIHUMIx sample S07 searched against the IGC reference catalog. The left panel shows results using Peptonizer2000 with corresponding probability scores, while the right panel displays results using unique peptides from Unipept. Correct taxonomic identifications are indicated by darker shades of blue.

#### Untailored Reference Databases Lead to Less Confident Results

To investigate the performance of the Peptonizer2000 with untailored reference databases, we searched the SIHUMIx sample S07 against two additional proteome reference databases. First, we used the Integrated Gene Catalogue (IGC) [https://www.nature.com/articles/nbt.2942] from the CAMPI PRIDE repository. This catalog includes reference proteomes for many genera commonly found in the human gut, including those present in the SIHUMIx sample, but may not have exact species matches.

The results, presented in Figure 5, also include species-level taxonomic identifications based on unique peptides generated by Unipept. Three species present in low abundance in SIHUMIx—*Lactoplantibacillus plantarium*, *Clostridium butyricum*, and *Bifidobacterium longum*—were not detected by either Peptonizer2000 or Unipept. The use of a larger and less specific database resulted in no unique peptides being identified for these species. Consequently, the number of peptide hits was too low to be included in the Peptonizer2000 graphical model. For the five detected taxa, Peptonizer2000’s probability scores and the number of unique peptides detected allowed accurate conclusions about the sample’s composition. All five correct taxa received the highest scores with Peptonizer2000. The next highest scoring taxon, *Blautia obeum*, did not appear in the top taxa of the Unipept analysis and can thus be excluded. Relying solely on Unipept would have made it more difficult to determine which taxa were truly present. To make a sound recommendation regarding a potential score cutoff, we computed precision, recall, and F1 score for taxa with at least one unique peptide, shown in Supplementary Figure S13. This leads us to a slightly more conservative score cutoff recommendation of *s* > 0.9 for species-level results and untailored references databases.

To simulate a scenario without prior knowledge of the microbial sample, we analyzed sample S07 from CAMPI using UniRef90.^46^ This large and unspecialized database significantly reduces the peptide identification rate, resulting in lower accuracy for taxonomic assignments. As anticipated, using such an extensive database impeded accurate taxonomic identification, this is linked to the known effect of results worsening with inflated database size. For SIHUMIx, a maximum of five correct taxa appear in the top 20 results.

#### Missing Taxa in the Taxonomic Reference Can Lead to Less Reliable Results

As many taxa present in real microbiomes, such as poorly sequenced, unculturable, or simply novel organisms, are likely to be absent from the UniProt database and therefore from our workflow, we simulated this by manually removing *Bacteroides thetaiotaomicron* at the species and genus level, *Lactiplantibacillus plantarum* at species level, and all SIHUMIx taxa at species level from the Peptonizer2000’s graph for the sample S07. The results are shown in Figure 6 and the removed taxa are indicated above each result. The analysis shows that, in the absence of the correct species or genus from the database used for taxonomic information, the Peptonizer2000 will assign very high probabilities to the most closely related taxa: When *Bacteroides thetaiotaomicron* is absent, multiple species from the genus *Bacteroides* are assigned very high probabilities. Similarly, when the whole *Bacteroides* genus is absent, many species from the next higher common taxonomic level, the *Bacteroidaceae* family, are detected as present. If many very closely related taxa, e.g., from the same genus, obtain high probability scores, users should consider the possibility that the organism present in their microbiome is not in the reference database.

**Figure 6:**
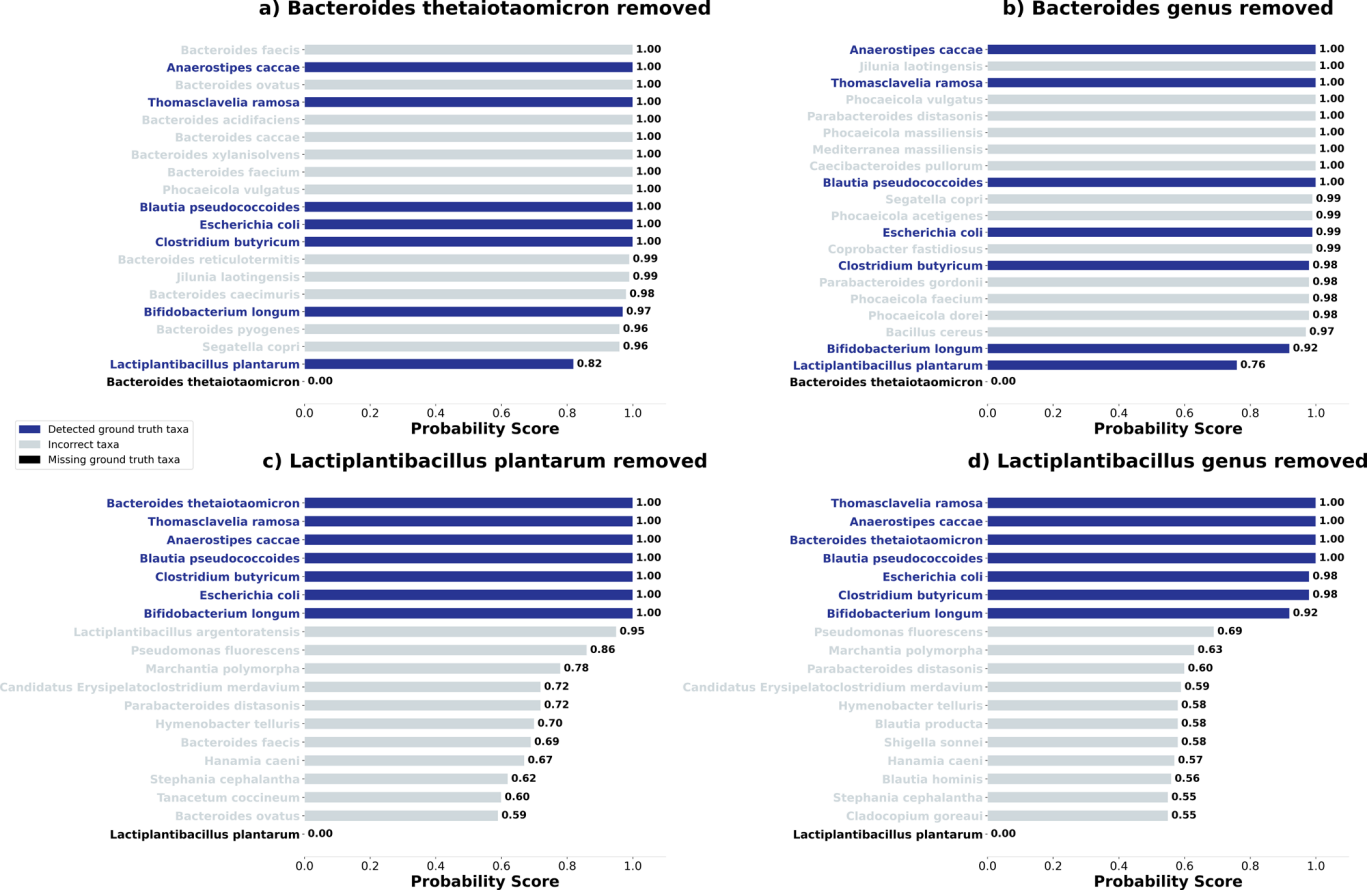
Taxonomic identification results for the Peptonizer2000 when selected taxa are missing from the Unipept database. The removed taxa are specified above each individual graph.

For species for which very few peptides were detected in the original sample, or where far fewer references are available, such as *Lactiplantibacillus plantarum*, a closely related species from the same genus gets identified as present. When the whole genus is absent, no taxon related to it is identified, which is due to the low number of peptides detected. Removing other taxa at species or family level, or even removing all SIHUMIx species, leads to similar conclusions. The results are shown in the Supporting Information.

#### Taxonomic Assignments in Fecal, Soil, and Ocean Samples

To evaluate the performance of the Peptonizer2000 on real microbiomes, we analyzed the fecal sample F07 from the CAMPI study, an ocean microbiome and a soil microbiome. We used the IGC gut reference database for the fecal sample and databases provided through PRIDE repositories for the soil sample (a sample-tailored database with NCBI and metagenomics assembly as source) and the ocean sample (a metagenomics-assembled database). We evaluated all samples at three taxonomic resolution levels: species and genus, for which the results are shown in the Supporting Information, and the one used in the original publication to discuss our results. For the fecal sample, this was family level, for the soil sample phylum, and for the ocean sample class. Figure 7A shows the Peptonizer2000 results at the family level with a probability score above 0.6, detecting 22 bacterial families. Overall, these results align with previous analyses of fecal samples in the CAMPI study^20^ and other human gut microbiome studies,^51,52^ demonstrating the ability of Peptonizer2000 to compute meaningful taxonomic probability scores at higher taxonomic levels.

**Figure 7:**
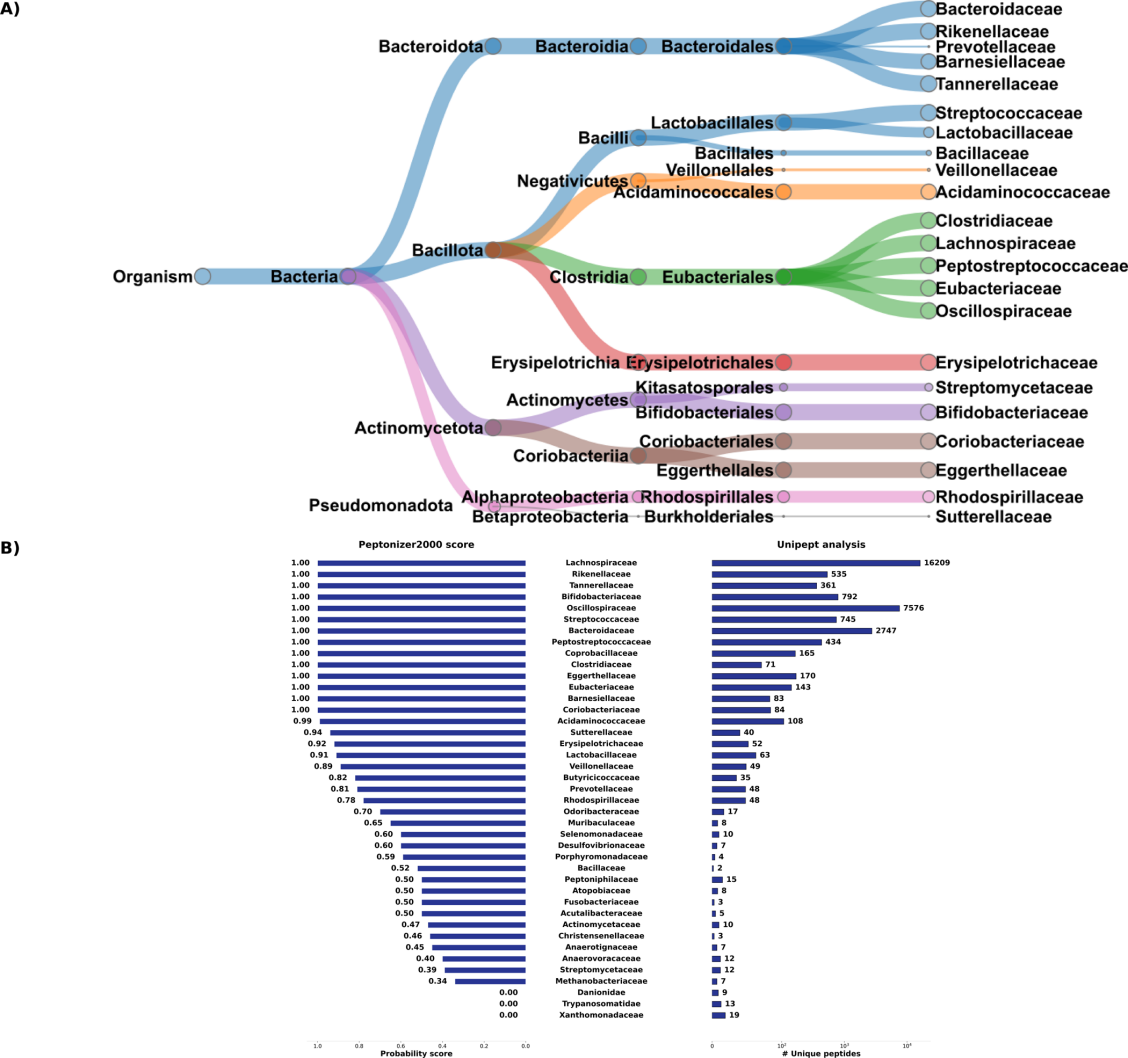
(A) Taxonomic tree view of the Peptonizer2000 results for fecal sample F07 from the CAMPI study, filtered with a probability score above 0.6. Node sizes are proportional to the probabilities attributed by the Peptonizer2000. (B) Bar plots without filtering of results showing the Peptonizer2000 analysis (left) and Unipept analysis (right).

The range of the number of unique peptides per family is very large, from 1 to 16209. All families with a very large number of unique peptides are also attributed very high probability scores by the Peptonizer2000. For families with lower numbers of taxonomic attributions, the Peptonizer2000 scores can hint toward the certainty of their presence. For example, the family *Sutterellaceae* has 40 unique peptides detected and a probability of presence of 0.94, and is therefore more likely to be present, which overlaps with previous analyses of this sample.^20^ *Bacillaceae* obtain a score of 0.52, even though only two unique peptides are detected. As this family of bacteria has been isolated from fecal samples before,^53^ they might truly be present in the fecal sample F07. Still, no further conclusions can be drawn since no ground truth is available.

The results for the soil and the ocean microbiomes are shown in the Supplementary Figures S15–S17. The results for both analyses are in accordance with the taxonomic identifications from the original publications, which highlights the applicability of the Peptonizer2000 to samples of diverse backgrounds. Few low-abundance taxa receive very low Peptonizer scores, which could be due to their poor representation in UniProt. In the future, the possibility to link probabilistic approaches like ours to more diverse databases can lead to additional accuracy improvements. The results shown for species and genus level show the possibility to provide taxonomic assignments at ranks lower than the original publications, although an assessment of accuracy would be speculative because of the absence of a ground truth.

### Parameter Choices for the Graphical Model Are Intuitively Explainable

For all analyses, the Peptonizer2000 was run for the same ranges of parameters α, γ, and β: α ∈ [0.7,0.8,0.9,0.99], β ∈ [0.2,0.3,0.4,0.5,0.6,0.7,0.8,0.9], and γ ∈ [0.1,0.3,0.5]. A detailed description of these parameters is found in the Methods section. A grid search then identifies the optimal parameter set for each sample. The parameter set selected for each sample is shown in Table 1.

**Table 1:** Parameters of the Noisy OR Conditional Probability Tables, α, β, and γ, Selected as Best Fitting for Each Sample

Sample	Origin/Composition	Reference database	α	β	γ
S03	CAMPI/SIHUMIx	tailored	0.99	0.7	0.5
S05	CAMPI/SIHUMIx	tailored	0.99	0.7	0.5
S07	CAMPI/SIHUMIx	tailored	0.99	0.7	0.5
S07	CAMPI/SIHUMIx	IGC gut	0.99	0.9	0.1
S08	CAMPI/SIHUMIx	tailored	0.99	0.6	0.5
S11	CAMPI/SIHUMIx	tailored	0.99	0.6	0.5
S11	CAMPI/SIHUMIx	tailored	0.99	0.6	0.5
F07	CAMPI/fecal	IGC gut	0.99	0.6	0.5
U1	Kleiner/28Mix	tailored	0.99	0.7	0.5
P1	Kleiner/28Mix	tailored	0.9	0.5	0.1
C1	Kleiner/28Mix	tailored	0.99	0.7	0.1

The parameter α represents the probability of detecting a peptide if the parent taxon is present. Given the large number of different peptides typically detected in metaproteomics, it makes sense that α ≥ 0.9 was selected for all samples. If a parent taxon is present, a peptide corresponding to it will likely be observed. This parameter choice aligns with parameter ranges identified as optimal in other studies using similar graphical models. For instance, for viruses, where the probability of detecting peptides is relatively low, the optimal α was found to be small.^31^ For more general protein inference, α was identified to be much higher.^35^

The parameter β represents the probability of detecting a peptide at random but can also be interpreted as the probability for a connection between a parent taxon and a detected peptide to be erroneously present. β ≥ 0.5 was identified for all samples, meaning the model assumes a high probability of a wrong peptide or a wrong connection between the peptide and its parent taxon. This observation agrees with the setup of our metaproteomic identification workflow: the peptides are queried against the whole taxonomy, and in the default settings, at least 150 taxa are included in the graphical model. Protein sequence homology, especially among housekeeping proteins, but also between closely related species, contributes to this high error probability. Many species and their connections to peptides not present in the sample will, therefore, still be present in the graphical model, which is reflected in the high error probability β. The parameter identified for γ varies: γ = 0.5 indicates no prior knowledge of taxa presence, which is common in metaproteomics. A lower γ suggests that when more than 150 species are included in the graph but only 30 species are actually present in a sample, most taxa in the graphical model are more likely to be absent than present.

Reducing the number of parameter combinations can greatly reduce the computational demands of the Peptonizer2000 workflow. Based on the observed parameters for the tested samples, we recommend including the following in the grid search for future metaproteomic analyses: α ∈ [0.85,0.9,0.99], β ∈ [0.5,0.6,0.7], and γ ∈ [0.1,0.3,0.5]. These choices represent reasonable defaults and reduce the grid search space to 27 parameter combinations.

### Reasonable Memory Use and Runtime

The Peptonizer2000 workflow consists of several steps, with the belief propagation being the most time-consuming. As of Unipept 5.0, the query time in Unipept is reduced to several minutes. The belief propagation algorithm runs until approximate convergence, and all parameter combinations can be run fully parallelized on multiple CPUs using Snakemake. If parameter sets need to be run sequentially, runtime will increase. The speed of convergence depends on the graph size, and on the distance of the initial beliefs of the graph and the final probability distribution. The worst case time complexity of the initialization of the messages for the belief propagation method is *O*(*n* ^2^), where *n* is the number of nodes in the graph. We benchmarked execution times using Intel Xeon E5-2650 v2 CPUs for different test samples, representing different graph sizes (Table 2).

**Table 2:** Benchmark of Runtime and Memory Use of the Peptonizer Workflow, across All Parameters Ranges, for Samples Producing Graphs of Different Sizes

Sample	# nodes (*N*) # edges (*e*)	CPU time (s) min–max	Memory (GB) min–max
S03	*N* = 48115, *e* = 197436	940–1453	2.5–2.6
S05	*N* = 54622, *e* = 239145	1612–2832	3.7–3.8
S11	*N* = 74434, *e* = 323624	2177–3426	4.9–5.1
F07	*N* = 30000, *e* = 111775	334–489	0.92–0.98
U1	*N* = 66289, *e* = 230546	581–916	1.8 −1.9

To take full advantage of parallelization, we recommend running the Peptonizer2000 in server environments.

## Discussion and Outlook

In this study, we introduced the Peptonizer2000, a graphical model-based workflow designed to determine the taxonomic composition of microbiome samples through metaproteomic analysis. The primary input is a file with one column for detected peptides and another for attributed scores. In contrast to previous methods for metaproteomic analysis, the Peptonizer2000 incorporates peptides and their scores from database searches into the workflow, using the scores to refine taxonomic assignments. Peptide data is processed through a newly developed Unipept API end point, which improves the taxon inference process. Using the input, Peptonizer2000 builds a graphical model of the joint probability distribution of peptides and associated taxa. The model is subsequently calibrated, and marginal probabilities for the presence of each taxon included in the graph are computed, offering a probabilistic estimate of taxonomic composition.

We have shown that the Peptonizer2000 generates reliable probability scores for metaproteomic samples of varying complexity. In particular, we highlighted the benefit of using the probability scores together with the number of unique peptides per taxon, as identified by Unipept, to refine the determination of taxa presence or absence. This can especially help to justify the imposition of thresholds such as “at least two unique peptides per taxon”, a heuristic very commonly used in metaproteomics. In addition, we showed that Peptonizer2000 can identify taxa, such as genera, with reasonable probability scores, even when no unique peptides are detected. This is achieved by integrating shared peptides into the graphical model, as demonstrated in a fecal microbiome sample analysis. This ability to infer the presence of taxa despite the absence of unique peptides highlights Peptonizer2000’s potential to improve the accuracy of taxonomic profiling in complex microbial communities.

Because MS/MS directly measures peptides and many are shared across homologous proteins and taxa, a peptide-centric formulation aligns naturally with the aims of the Peptonizer2000: the graphical model explicitly retains shared peptides and propagates their evidence into calibrated posterior probabilities for taxa. This avoids premature aggregation and the nonidentifiability that protein inference can introduce in complex communities. Consistent with recent reports, peptide-centric analyses enhance taxonomic assignment and uncover richer functional signal relative to protein-level summarization.^19^ Importantly, the Peptonizer2000’s inference algorithm is, in principle, applicable at the protein (or protein-group) level by substituting peptide nodes with protein nodes and redefining the mapping to taxa, at the expense of the additional assumptions required for protein grouping.

In the analysis of more complex lab-assembled mixtures, the Peptonizer2000 computed robust probability assignments for most species present but was less able to resolve very closely related species. Including the number of unique peptides attributed by Unipept can help resolve such ambiguities. In the future, probabilistic quantification algorithms incorporating spectral abundance measures or MS1-based quantification could further improve taxonomic identification and propagate uncertainty-aware scores to quantification.

As the Peptonizer2000 computes a probability distribution, imposing an exact threshold posterior probability to determine the presence or absence of a taxon is not justifiable. However, based on the previously described results and analysis of F1 scores, we can estimate approximate threshold probabilities. For low-complexity samples, such as SIHUMIx, the threshold should be above a score of 0.9 to safely exclude false positives and can be relaxed to a score of 0.8 if we include the criterion of detecting at least one unique peptide. For medium-complexity samples, such as lab-assembled mixtures or samples analyzed with untailored but not generalist reference databases (e.g., SIHUMIx searched against IGC), the threshold should be around 0.8. Based on the F1 score, we cannot recommend imposing a minimum of unique peptides detected for a taxon, but researchers can use this to increase results confidence. If many very closely related taxa, e.g., from the same genus, obtain high probability scores, users should consider the possibility that the organism present in their microbiome is not present in the reference database. For high-complexity samples or those analyzed with generalist reference databases (e.g., fecal samples searched against UniProtKB), we would not recommend any specific threshold value. The variability in sample complexity and the chosen database significantly impact the determination of appropriate threshold values, highlighting the need for careful consideration in each specific context. Alternatively, future developments in metaproteomic analysis could evolve from the currently accepted practices of setting false-discovery rates and arbitrary cutoffs toward more sensitive data analysis methods able to handle the unavoidable uncertainties of any data acquisition method and produce probabilistic error estimates.^54^

Currently, we recommend running the Peptonizer2000 in server environments due to its computational demands. In the future, several algorithmic improvements will significantly speed up the workflow.

For computing taxonomic probabilities, we currently use zero-lookahead belief propagation, which has been shown to be up to five times faster than standard belief propagation.^37^ Despite this improvement, our method remains dependent on graph size, which practically limits the number of taxa that can be included in the graphical model. To address this limitation, graph clustering approaches through community detection^55^ could be employed in the future. These approaches substantially reduce graph size and, consequently, computation time by breaking up the graph of peptides and taxa into smaller, densely connected communities. Additionally, this would enable fully parallelized computation of taxon posteriors for each community, further enhancing efficiency.

To streamline access to Peptonizer2000 for researchers, we plan to integrate it into the Unipept web application (https://unipept.ugent.be/). This integration will enhance the existing capabilities for taxonomic analysis based on unique peptides while also providing a more user-friendly web interface.

It is important to note that Peptonizer2000 relies heavily on the peptides detected through MS/MS experiments and downstream bioinformatic analyses. Ongoing research and advancements in peptide detection, such as data-independent acquisition methods for metaproteomics^21,29^ and the development of database search engines capable of effectively resolving chimeric spectra,^56^ can be expected to translate into more accurate and robust taxonomic identifications.

Lastly, the Peptonizer2000 is designed to work directly with Unipept API outputs once a peptide list is provided. In theory, the Peptonizer2000’s graphical model can be used with any reference database, which was the case, for example, for PepGM,^31^ that uses a curated viral reference from NCBI as input database. The Peptonizer2000 focuses specifically on application ease for nonbioinformaticians and speed and, therefore, is intrinsically linked to Unipept. This, however, has the natural drawback of limiting users to species references in UniProt. If users have prior knowledge about sample composition, they can limit the taxonomic range queried, which can improve results. We plan to provide a more generalized, stand-alone version of the belief propagation algorithm in the future, so that users will be able to use it in combination with more domain-specific databases such as MGnify^57^ or GTDB,^58^ and include it in varied workflows.

Looking ahead, these advancements will not only improve the speed and scalability of the Peptonizer2000 but also broaden its applicability to more complex and diverse data sets within the metaproteomics community. This progress will enable researchers to gain deeper insights from their data, ultimately advancing the understanding of microbial communities, including the identification and functional roles of their key players.

## Supplementary Material



## Notes

Table S1: List of Python packages used in the workflow. Tables S2–S4: The distribution of peptides in the Unipept database. Table S5: Composition of U1, C1, P1. Tables S6–S7: The species in the soil and ocean microbiomes and whether they were detected by the Peptonizer2000. Figure S1: LCA rank vs peptide length for the SIHUMIx s5 data set, demonstrating that longer peptides provide higher taxonomic specificity. Figure S2: LCA rank vs peptide length for the Kleiner U1 data set, showing the correlation between sequence length and taxonomic resolution. Figure S3: Taxonomic identification and probability scores for SIHUMIx sample s3 compared to Unipept unique peptide results. Figure S4: Comparison of Peptonizer2000 results and Unipept unique peptide identifications for SIHUMIx sample s5. Figure S5: Identification results and confidence scores for SIHUMIx sample s8 vs standard Unipept mapping. Figure S6: Precision, recall, and F1 score analysis for sample S07 to identify the optimal scoring threshold. Figure S7: Threshold analysis (Precision/Recall/F1) for SIHUMIx sample S11. Figure S8: Performance metrics for sample S11 when restricted to taxa with at least one unique peptide. Figure S9: Investigation of how varying the number of taxa in the graphical model impacts identification accuracy for sample S07. Figure S10: Precision, recall, and F1 score threshold analysis for the complex lab-assembled sample U1. Figure S11: Identification results for the equal cell content mixture (C1) compared to Unipept. Figure S12: Comparison of Peptonizer2000 and Unipept results for the equal protein content mixture (P1). Figure S13: Performance metrics for sample S07 when searched against the Integrated Gene Catalog (IGC) database. Figure S14: Identification results for sample S07 using the UniRef90 database. Figure S15: Genus and species-level taxonomic identification for a diverse human fecal sample (F07). Figure S16: High-level taxonomic identification (class and phylum level) for environmental ocean and soil samples. Figure S17: Top-scoring genus and species-level identifications for the ocean sample. Figure S18: Top-scoring genus and species-level identifications for the soil sample (PDF)
