# Supplementary material for: The Peptonizer2000: Bringing Confidence to Metaproteomics
Source: J Proteome Res. 2026 Mar 4;25(4):1831–45. doi: 10.1021/acs.jproteome.5c00567 (PMC13054872; doi:10.1021/acs.jproteome.5c00567)
Supplement: Supplementary file 1 [file pr5c00567_si_001.pdf]

Supplementary material for 'The Peptonizer2000: bringing confidence to metaproteomics'

Tanja Holstein,[†,‡,¶,§,‖] Pieter Verschaffelt,[†,‡] Tim Van Den Bossche,[†,‡] Simon Van de Vyver,[⊥] Lennart Martens,[†,‡,§,‖] Bart Mesuere,[⊥] and Thilo Muth[*,¶]

† *VIB-UGent center for Medical Biotechnology, VIB, Belgium*

‡ *Department of Biomolecular Medicine, Ghent University, Belgium*

¶ *Data Competence Center MF 2, Robert Koch Institute, Berlin, Germany*

§ *BioOrganic Mass Spectrometry Laboratory (LSMBO), IPHC UMR 7178, University of Strasbourg, CNRS, Strasbourg, 67000, France*

‖ *Infrastructure Nationale de Protéomique ProFI FR2048, Strasbourg, 67087, France*

⊥ *Department of Applied Mathematics, Computer Science and Statistics, Ghent University, Belgium*

E-mail: mutht@rki.de

List of Figures

S1 LCA vs peptide length for SIHUMIx s5 . . . . S7

S2 LCA vs peptide length for Kleiner U1 . . . . S8

S3 Taxonomic identification of SIHUMIx s3 . . . . S10

S4 Taxonomic identification of SIHUMIx s5 . . . . S11

S5 Taxonomic identification of SIHUMIx s8 . . . . S11

S6 Threshold analysis for sample S07 . . . . S12

S7 Threshold analysis for sample S11 . . . . S13

S8 Combined threshold analysis for S11 . . . S14
S9 Impact of taxa count on S07 results . . . S15
S10 Threshold analysis for sample U1 . . . S16
S11 Taxonomic identification of C1 . . . S17
S12 Taxonomic identification of P1 . . . S18
S13 S07 identification against IGC . . . S19
S14 S07 identification against UNIREF90 . . . S20
S15 Taxonomic identification of fecal sample F07 . . . S21
S16 Ocean and soil identification comparison . . . S22
S17 Detailed ocean identification . . . S23
S18 Detailed soil identification . . . S24

List of Tables

S1 Libraries used for Peptonizer2000 . . . S3
S2 Peptide-protein associations in Unipept . . . S4
S3 LCA of excluded peptides . . . S5
S4 Species-specific peptides with high protein mapping . . . S6
S5 Composition of samples U, C, and P . . . S9
S6 Taxa detection in soil sample . . . S22
S7 Taxa detection in ocean sample . . . S23

Additional information regarding the Peptonizer2000 workflow

List of packages used

Table S1: Packages used in the Peptonizer2000

Package name	reference
Ete3	[1]
pandas	[2]
numpy	[?]
networkx	[3]
scipy	[4]
Biopython	[5]
matplotlib	[6]
seaborn	[7]

Taxonomic rank of peptides and proteins excluded by the Peptonizer

The Peptonizer2000 excludes peptides from its Unipept query that map to more than 10000 proteins. In the following, we describe which peptide taxon mappings they exclude and why this leads to no, or only a negligible reduction in taxonomic resolution.

Every peptide sequence in the Unipept database is associated with one or more proteins in which the sequence occurs. The amount of proteins that a peptide is associated with can differ drastically. The following table presents an overview of how many peptides are associated with n or more proteins:

Table S2 shows that the amount of peptides associated with a large number of proteins decreases rapidly. There are only a little over 13000 peptides that occur in 10000 or more proteins. For these peptides, we expect the lowest common ancestor to be very generic, as they might, e.g., originate from housekeeping proteins. In the following, we analyse how far

Table S2: number of peptides associated to n or more Proteins in the Unipept database

# associated proteins n	# peptides
≥ 1	1 342 470 764
≥ 2	355 979 324
≥ 10	38 697 210
$\geq 10^2$	2 921 879
$\geq 10^3$	217 922
$\geq 10^4$	13 008
$\geq 10^5$	118
$\geq 10^6$	0

this expectation holds.

The 13000 peptides mapping to 10000 or more proteins are extracted and queried for their LCA. The results are shown in table S3.

Indeed, about 94% of the excluded peptides have 'root' as their LCA and therefore carry no taxonomic information. It is therefore sound to exclude these peptides from the Peptonizer analysis.

Interestingly, there are the 200 peptide sequences with an LCA at the species rank. Table S3 shows which species the peptide with more than 10000 proteins associated, but with high taxon specificity, map to.

The majority of these species are viral. For most viruses shown, such as HIV or influenza, research is intensive and enormous amounts of different strains exist. Many references are uploaded to the Uniprot database by scientists, explaining why there are 200 peptides that occur in 10000 or more proteins and that still have an LCA annotated at the species rank. On a side note, this showcases the bias towards model organisms and certain species or strains in public proteome databases.

Table S3: LCA of the 13000 peptides mapping to more than 10000 proteins.

NCBI taxonomy rank	Peptides
root	12 369
superkingdom	43
kingdom	16
subkingdom	0
superphylum	0
phylum	8
subphylum	7
superclass	1
class	18
subclass	1
superorder	0
order	0
infraorder	1
superfamily	0
family	2
subfamily	0
tribe	1
subtribe	0
genus	55
subgenus	0
species group	0
species subgroup	0
species	200
subspecies	0
strain	1
varietas	0
forma	0

Table S4: Amount of species-specific peptides per species that map to more than 10000 proteins.

# Peptides	LCA
119	Alphainfluenzavirus influenzae
32	Human immunodeficiency virus
14	Hepatitis B virus
9	Betainfluenzavirus influenzae
4	Orthoflavivirus denguei
3	Simian immunodeficiency virus
1	Alcidodes juglans
1	Bacillus subtilis
1	Bacteroides thetaiotaomicron
1	Cannabis sativa
1	Capsicum baccatum
1	Echinocucumis hispida
1	Geissoloma marginatum
1	Homo sapiens
1	Human immunodeficiency virus
1	Kalanchoe fedtschenkoi
1	Leucosceptrum canum
1	Loxia curvirostra
1	Marinilactibacillus piezotolerans
1	Melanocenchris jacquemontii
1	Merops nubicus
1	Morbillivirus hominis
1	Phalaenopsis pulcherrima
1	Phormidesmis priestleyi
1	Rhodobacter maris

Peptide length and taxonomic specificity

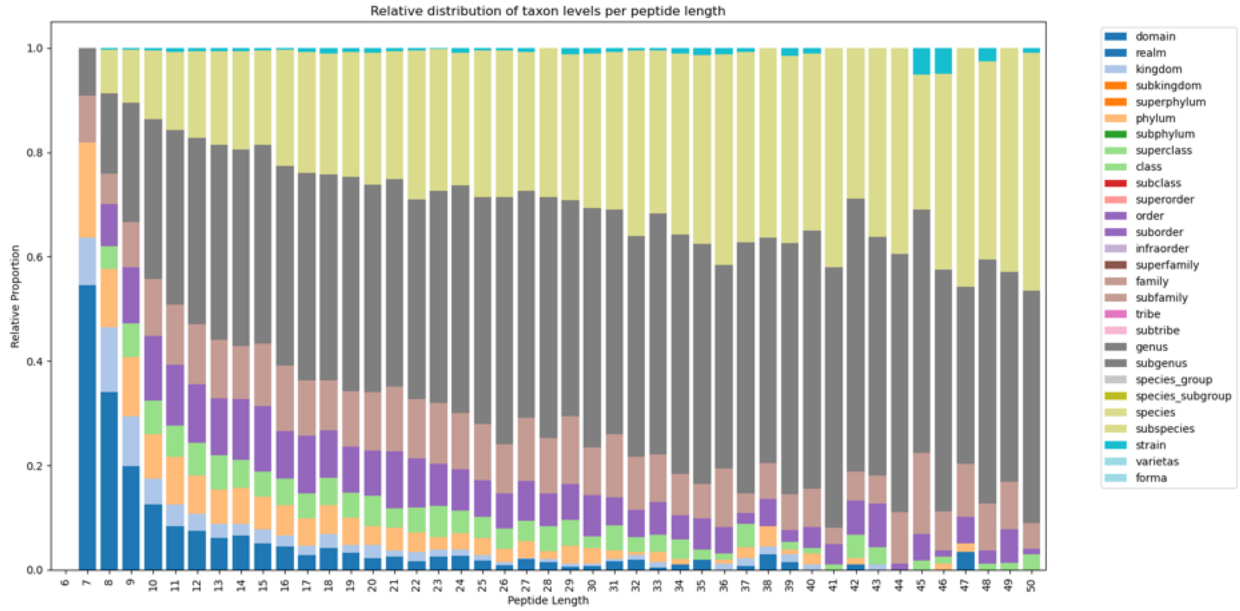

Figure S1: LCA versus peptide length for the peptides included in the s5 SIHUMIx dataset. Longer Peptides are more speific to lower taxonomic ranks, as they have more LCAs at species or family level.

We conducted a brief investigation of taxonomic specificity related to peptide length, shown for samples U5 and U1 in figures S1 and S2 respectively. To this end, we mapped the LCA against the peptide length- As expected, longer peptides tend to have a lower rank as LCA - the longer the peptide, the more likely it is to be species specific. This attribute is automatically taken into account by the graph structure at the base of the Peptonizer2000's probability distribution.

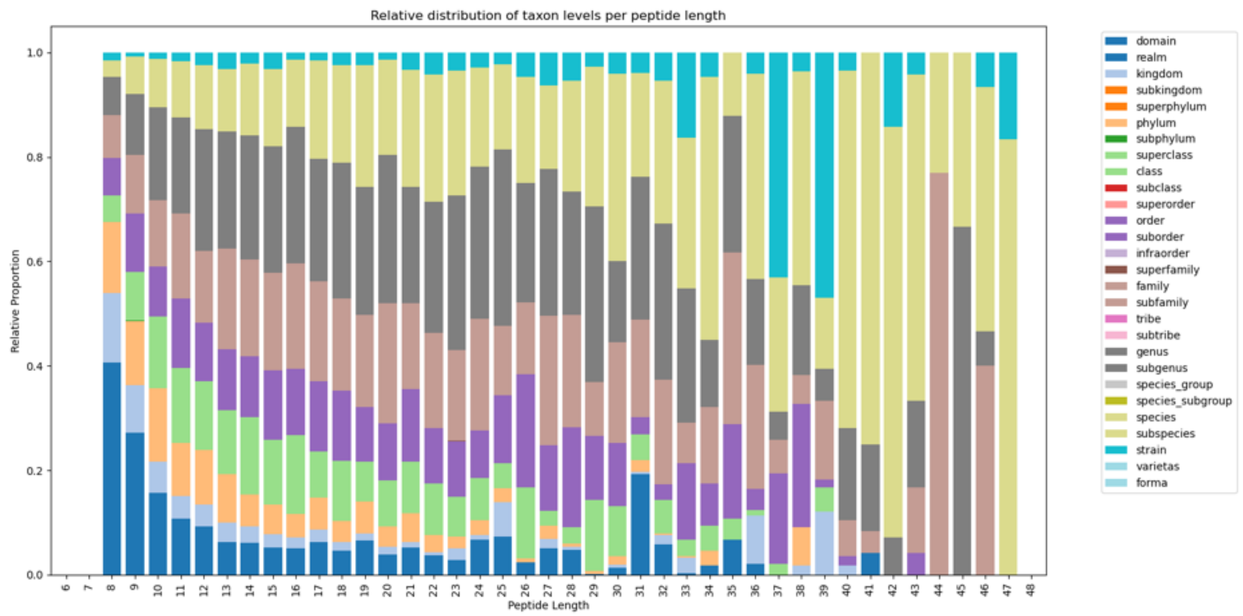

Figure S2: LCA versus peptide length for the peptides included in the U1 Kleiner dataset. Longer Peptides are more speific to lower taxonomic ranks, as they have more LCAs at species or family level.

Composition of the samples C1, P1, U1

The composition of the lab assembles samples C1,P1 und U1 was described in.[8] For convenience, we resume the composition of the three samples in the table below.

Table S5: Taxonomic composition of the three lab assembled communities U, C and P. Units are in μg

**Species**	U	C	P
Agrobacterium tumefaciens	4186.28	361.51	642.7
Alteromonas macleodii	707.47	214.38	642.7
Bacillus subtilis	583.83	717.06	642.7
Burkholderia xenovorans	321.37	69.86	642.7
Chlamydomonas reinhardtii	2962.73	6583.85	642.7
Chromobacterium violaceum	933.08	741.72	642.7
Cupriavidus metallidurans	11504.98	216.26	642.7
Desulfovibrio vulgaris	701.58	0.00	0
Escherichia coli	4290.54	319.24	642.7
Nitrosomonas europaeae	60.58	0.00	0
Nitrosomonas ureae	402.68	0.00	0
Nitrososphaera viennensis	607.41	27.61	642.7
Nitrosospira multiformis	155.16	0.00	0
Paracoccus denitrificans	683.66	198.74	642.7
Phage ES18	65.55	45.20	62
Phage F0	65.28	5.58	62
Phage F2	62.04	62.04	62
Phage M13	109.02	109.02	62
Phage P22	78.77	0.93	62
Pseudomonas denitrificans	2128.51	551.43	642.7
Pseudomonas fluorescens	4964.22	738.72	642.7
Pseudomonas pseudoalcaligenes	863.80	716.25	642.7
Rhizobium leguminosarum bv. viciae 3841	680.47	348.96	642.7
Rhizobium leguminosarum bv. viciae VF39	1671.03	185.67	642.7
Roseobacter sp. AK199	1183.43	408.08	642.7
Salmonella enterica typhimurium (3 strains combined)	25037.78	1123.11	1928.1
Staphylococcus aureus ATCC 13709	715.15	30.05	642.7
Staphylococcus aureus ATCC 25923	1216.31	24.04	642.7
Stenotrophomonas maltophilia	5946.27	448.44	642.7
Thermus Thermophilus	1245.63	386.84	642.7

Additional Results plots

SIHUMIx samples

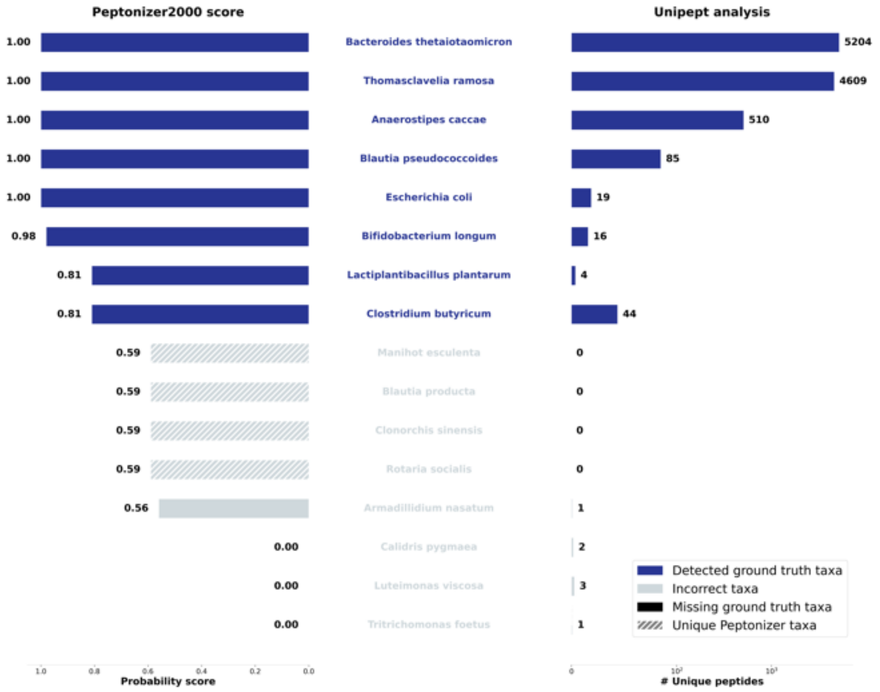

Figure S3: Results for the taxonomic identification of the SIHUMIx sample s3 using the Peptonizer2000, left, with corresponding probability scores, and using unique peptides from Unipept, right. Correctly identified taxa are shaded in a darker blue.

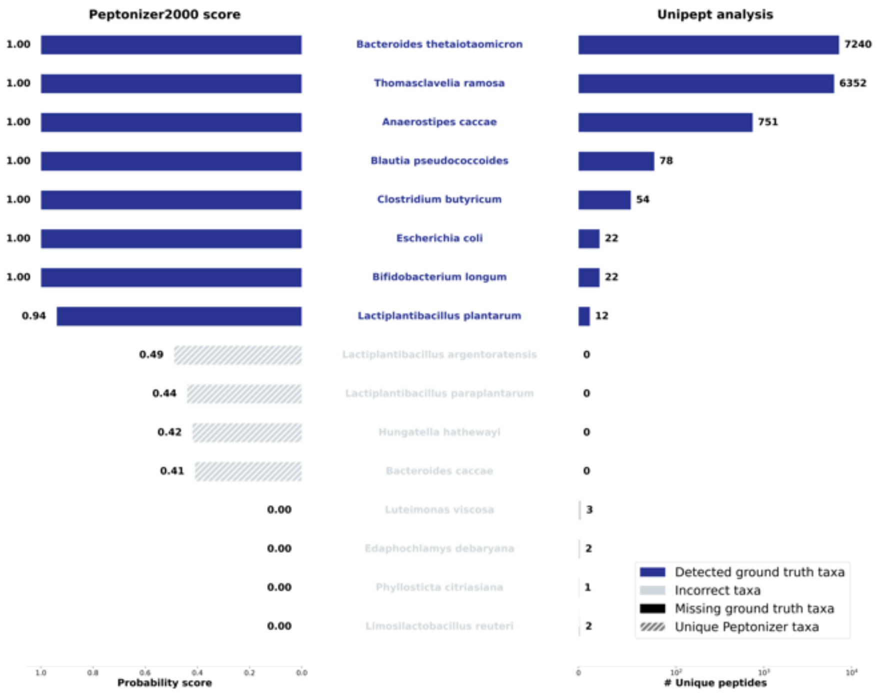

Figure S4: Results for the taxonomic identification of the SIHUMIx sample s5 using the Peptonizer2000, left, with corresponding probability scores, and using unique peptides from Unipept, right. Correctly identified taxa are shaded in a darker blue.

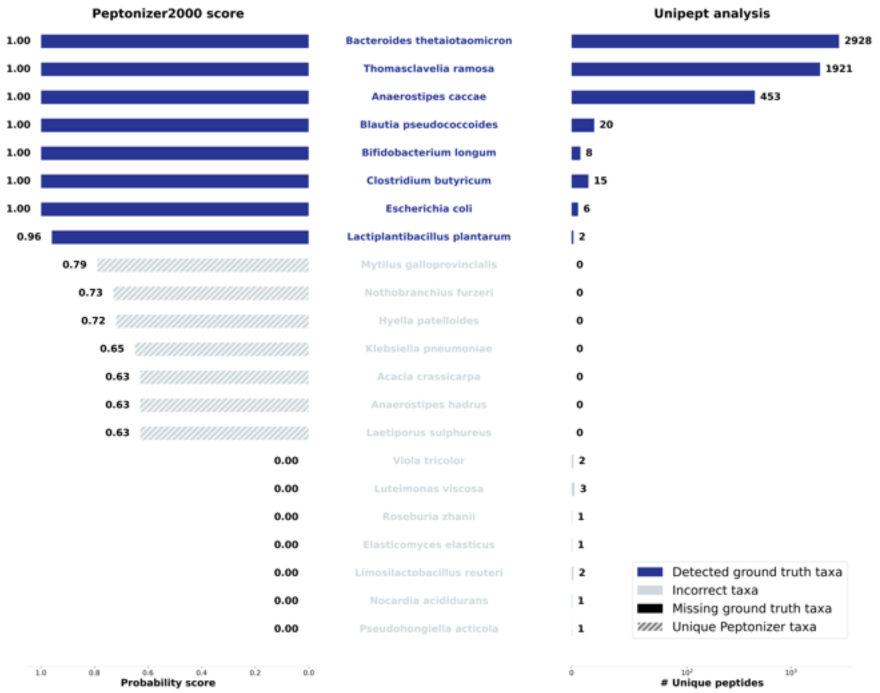

Figure S5: Results for the taxonomic identification of the SIHUMIx sample s8 using the Peptonizer2000, left, with corresponding probability scores, and using unique peptides from Unipept, right. Correctly identified taxa are shaded in a darker blue.

Threshold analysis for SIHUMIx

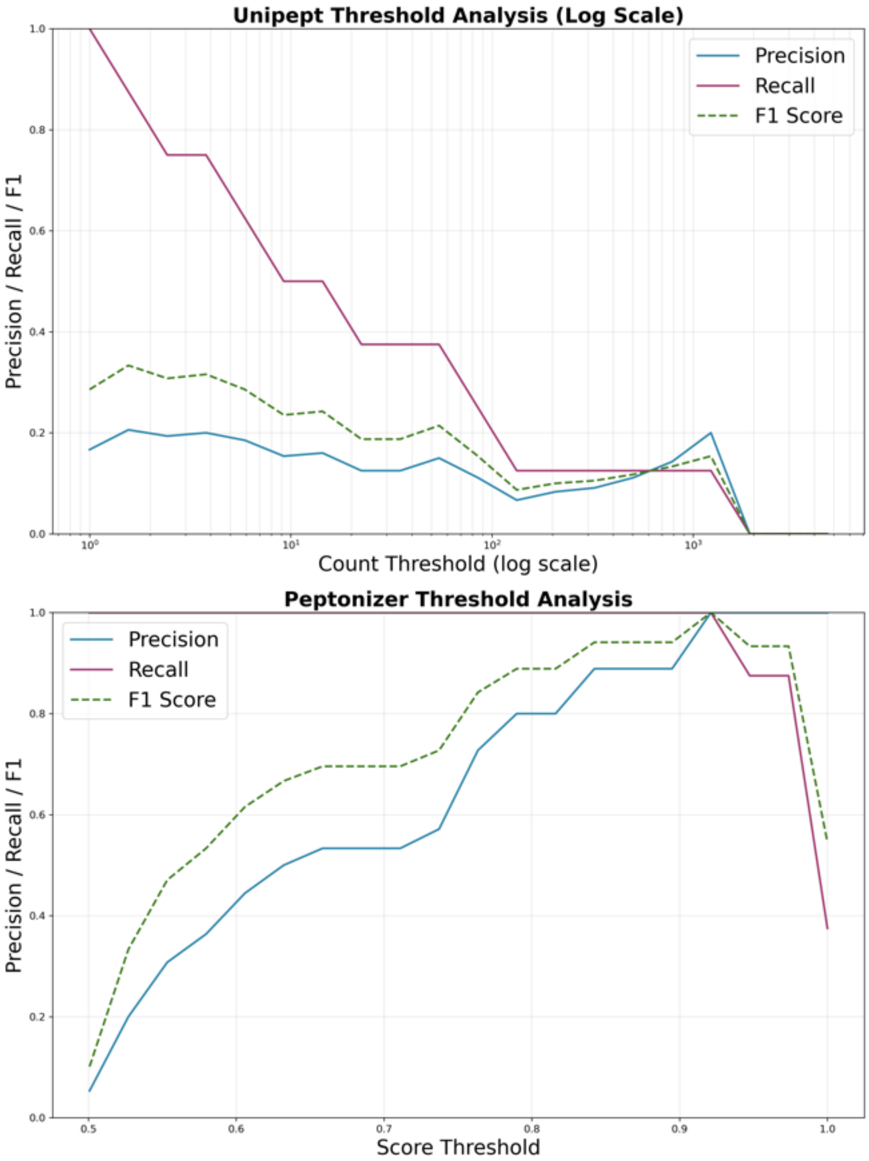

Figure S6: Precision, recall and F1 score for both Unipept and the Peptonizer for the sample S07. An optimal Peptonizer score seems to be around ≈ 0.9.

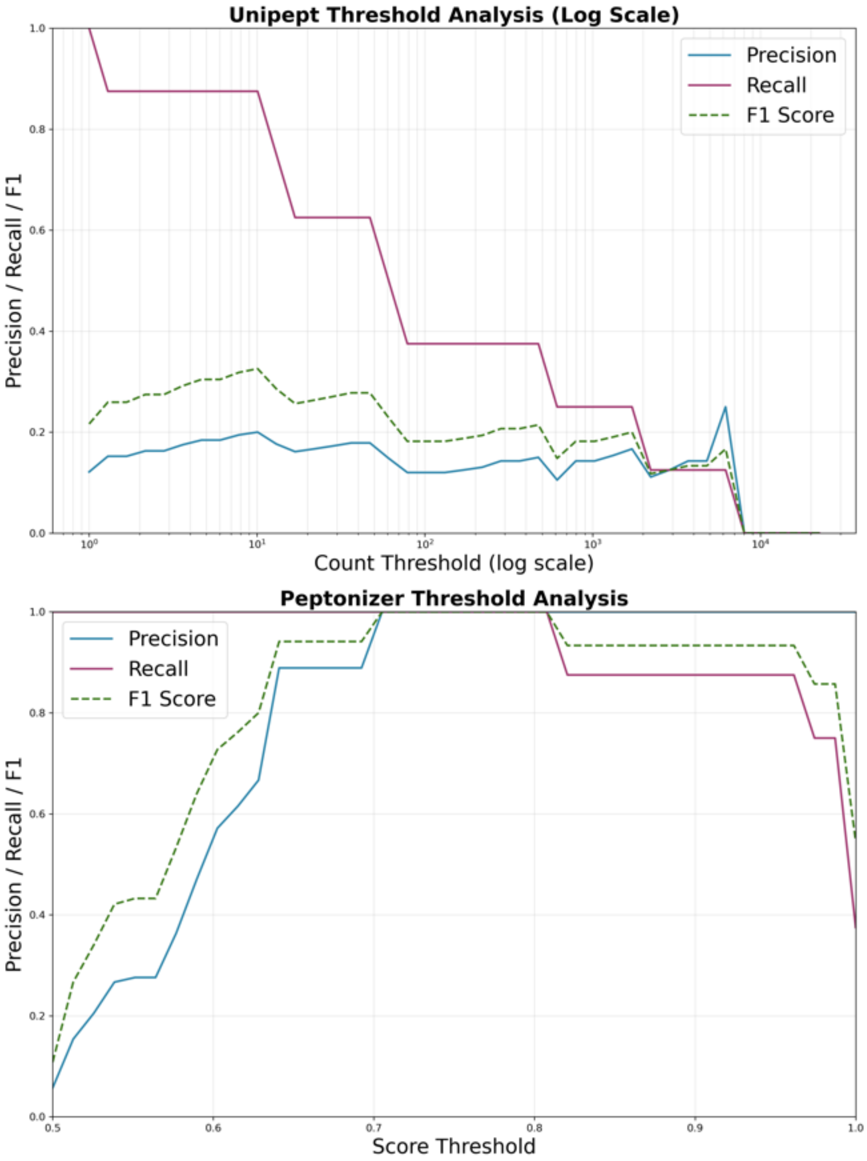

Figure S7: Precision, recall and F1 score for both Unipept and the Peptonizer for the sample S11. An optimal Peptonizer score seems to be around ≈ 0.9.

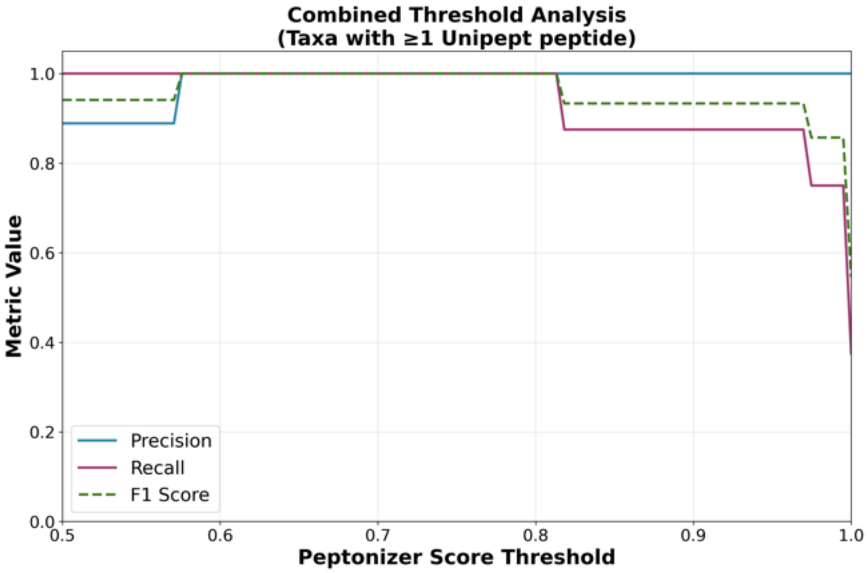

Figure S8: Precision, recall and F1 score the Peptonizer, including only taxa with at least one unique peptide, for the sample S11. An optimal Peptonizer score seems to be between 0.6 and 0.8.

Effect of number of included taxa

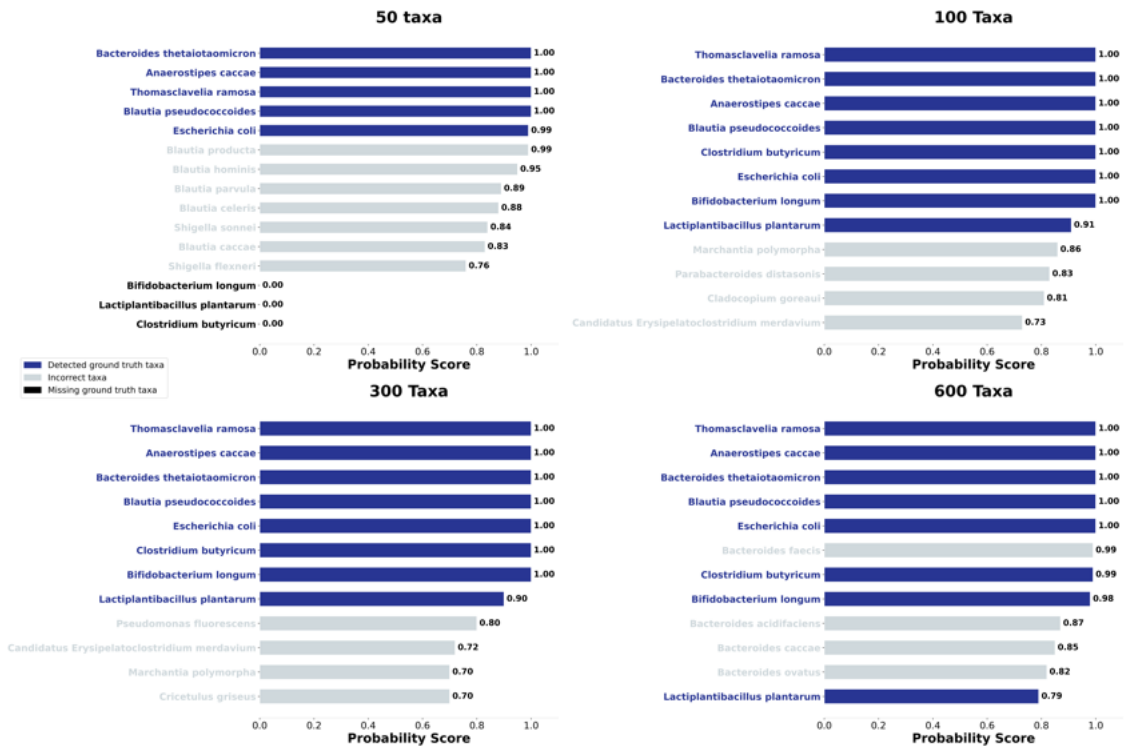

Figure S9: Investigation of Peptonizer results with different numbers of taxa included in the graph. the number is specified on top of each results plot. Results shown are for the sample S07.

The default setting for the number of taxa included in the graphical model is 150, and taxa are selected according to a weighted number of PSMs scheme as described in the main section of the manuscript. The number of taxa included does affect the results of the Peptonizer identification: if too few taxa are included, some actually present species could be excluded from the results, as can be seen for the plot with 50 taxa in Figure S9. If a very large number of taxa are included, some species that have many hits hits because they have many proteins present in Uniprot might be overrepresented, as is the case for *Bacteroides* species in the 600 taxa analysis. Overall, a default value of 150 taxa represent a reasonable balance between runtime and results accuracy. This parameter can be easily changed by users who which to explore microbiomes that they expect to be much more complex.

More complex samples

Threshold analysis for sample U1

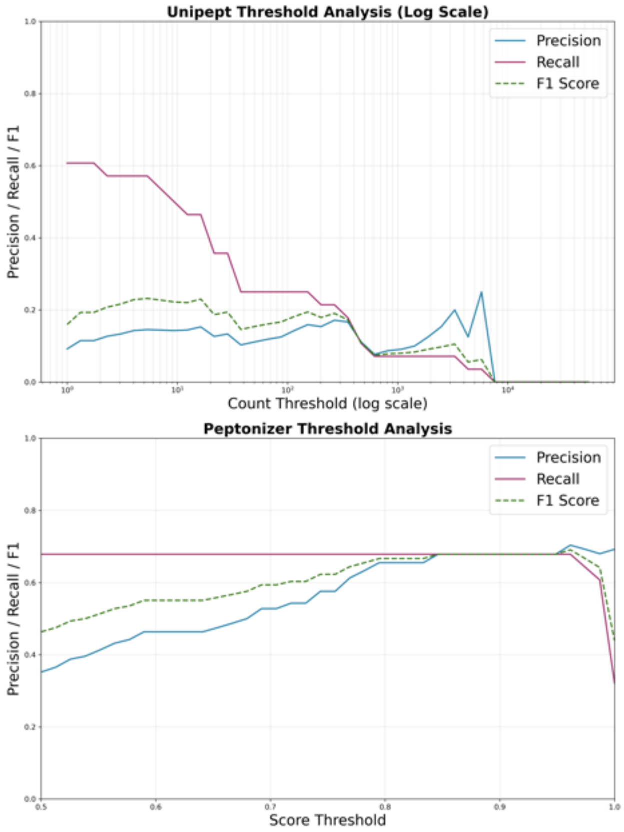

Figure S10: Precision, recall and F1 score the Peptonizer, including only taxa with at least one unique peptide, for the sample U1. An optimal Peptonizer score seems to be between 0.85 and 0.95.

Lab assembled mixtures C1 and P1

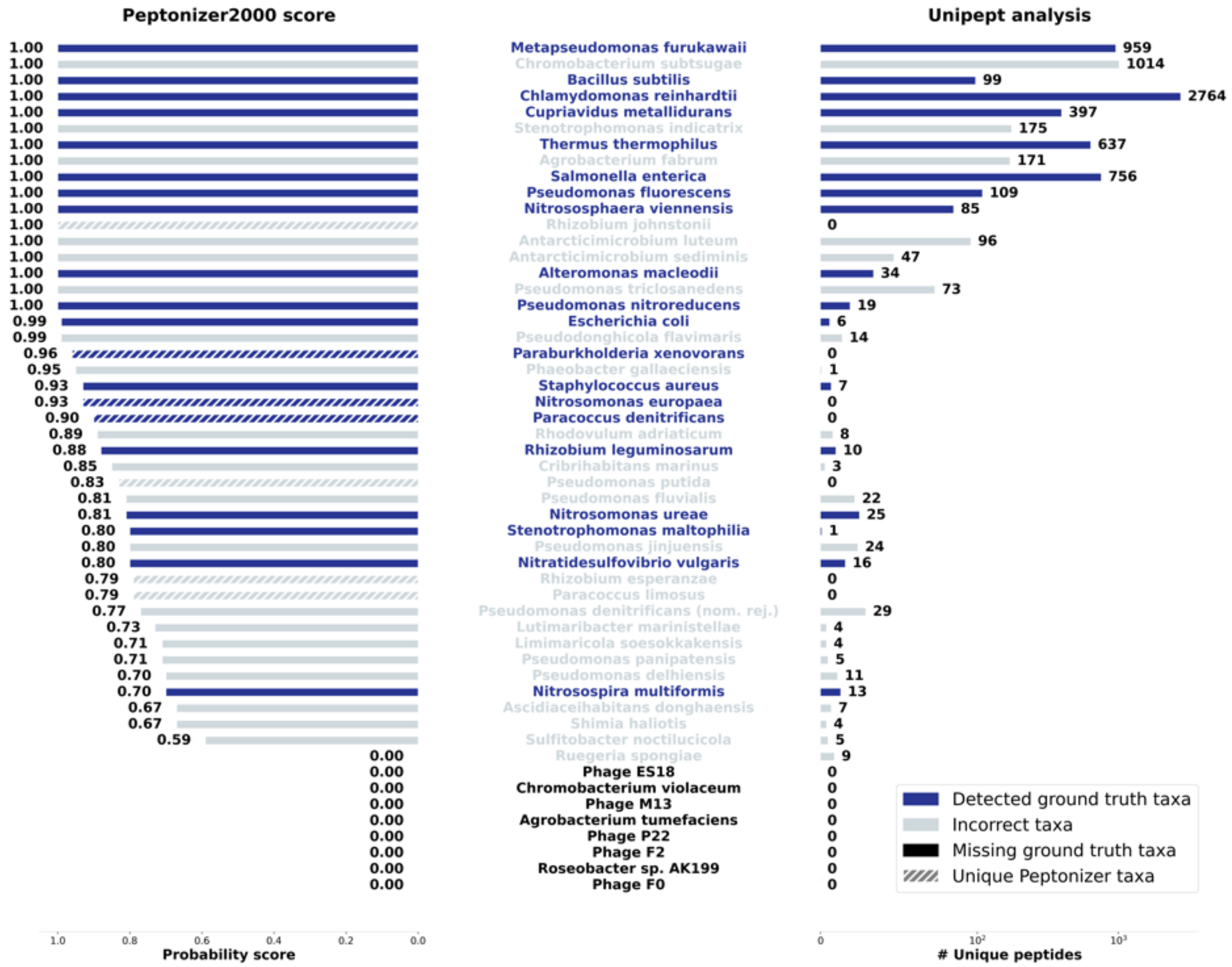

Figure S11: Results for the taxonomic identification of the lab assembled mixture with equal cell content C1 using the Peptonizer2000, left, with corresponding probability scores, and using unique peptides from Unipept, right. Correctly identified taxa are shaded in a darker blue.

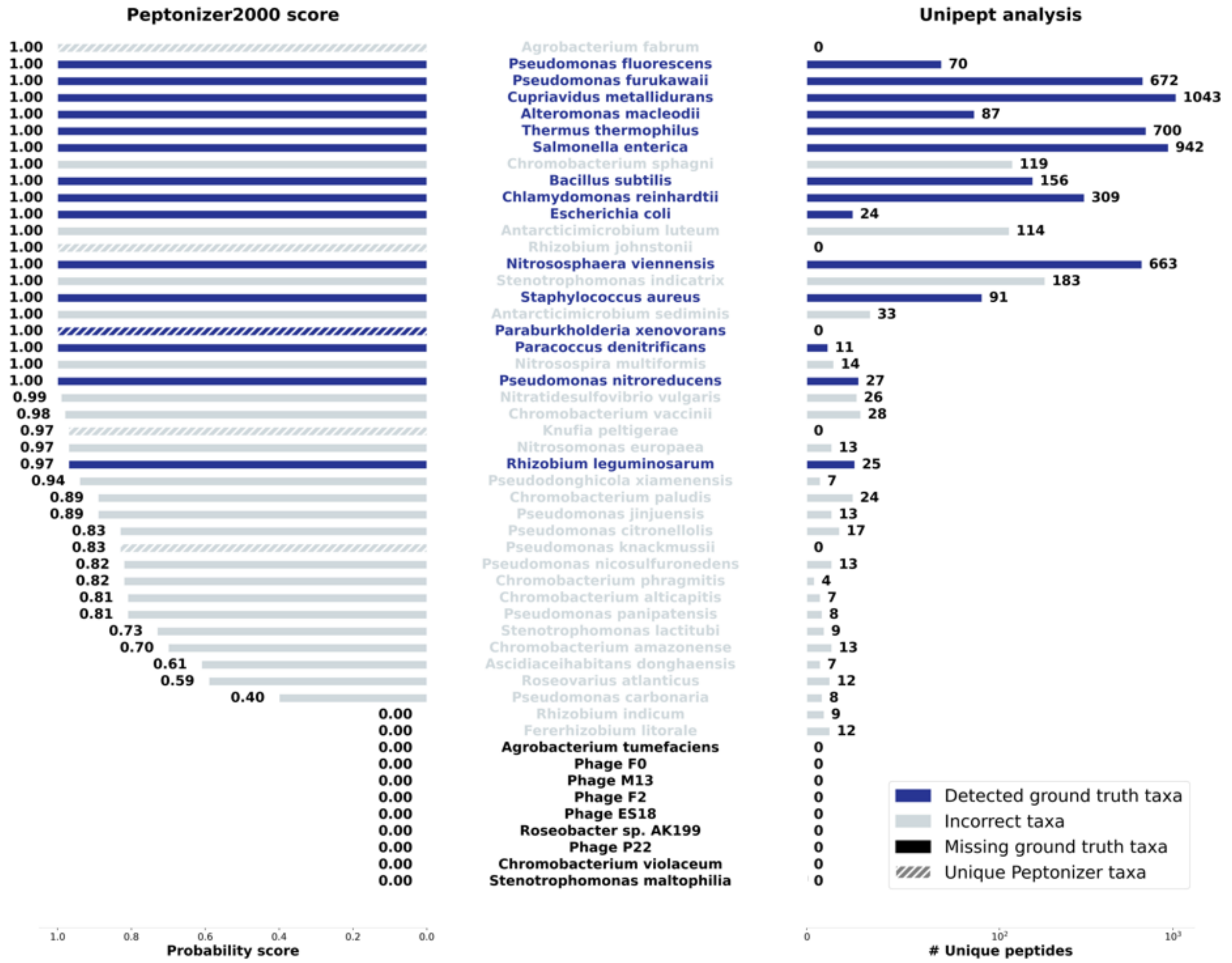

Figure S12: Results for the taxonomic identification of the lab assembled mixture with equal protein content P1 using the Peptonizer2000, left, with corresponding probability scores, and using unique peptides from Unipept, right. Correctly identified taxa are shaded in a darker blue.

Search against IGC and UNIREF90

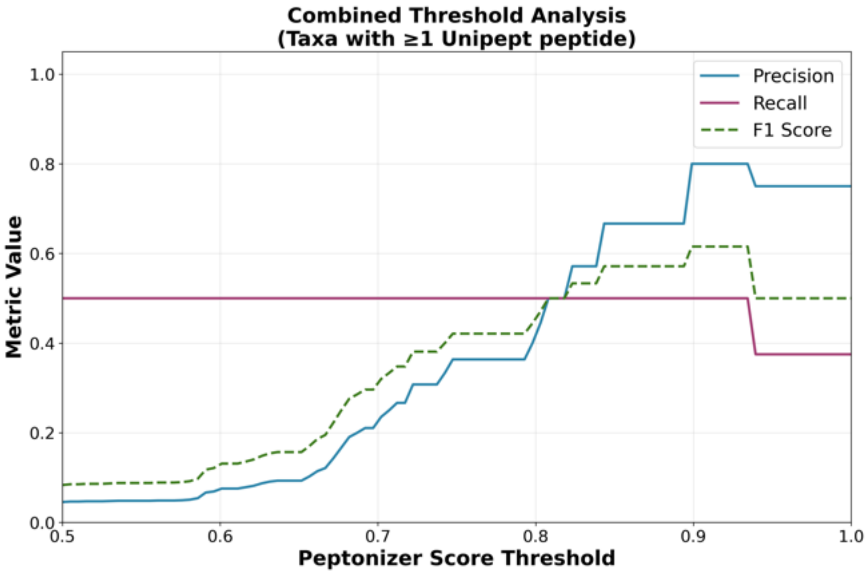

Figure S13: Precision, Recall and F1 score for the taxonomic identification of the SIHUMIx sample S07 searched against IGC. Only taxonomic identifications with at least one unique peptide are taken into account.

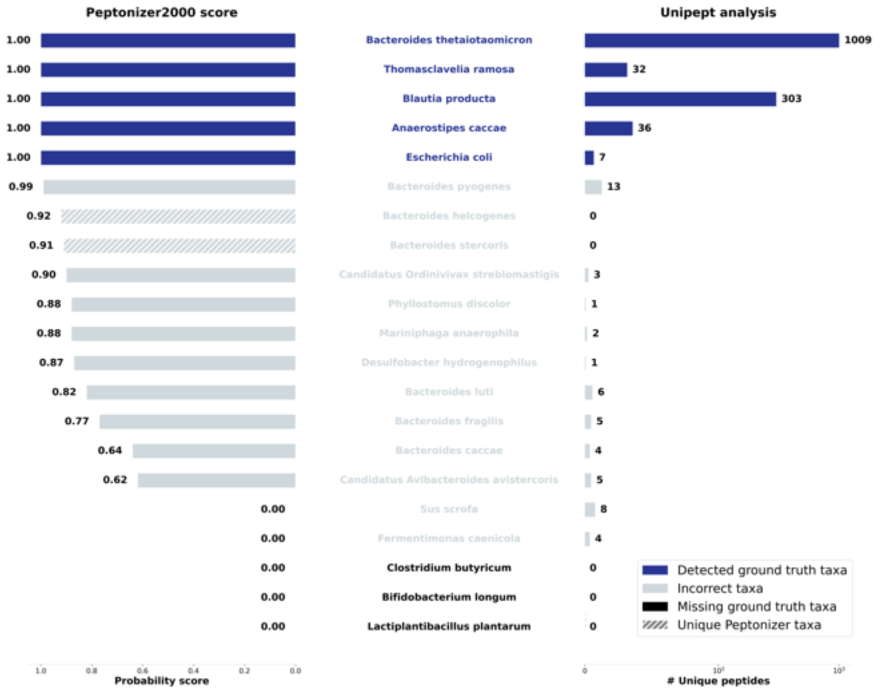

Figure S14: Results for the taxonomic identification of the SIHUMIx sample S07 searched against UNIREF90, using the Peptonizer2000, left, with corresponding probability scores, and using unique peptides from Unipept, right. Correctly identified taxa are shaded in a darker blue.

Results for more diverse microbiomes

In Figure S15, we examined the results from Peptonizer2000 at both genus and species levels. Although the Peptonizer2000 identified many species as present, human gut microbiomes are rarely analyzed with comprehensive species-level resolution. Therefore, we focus on discussing the genus-level results shown in Figure S15. All genera with high scores are know to be present in the human gut. Examples include *Anaerobutyricum*[9] and *Fusicatenibacter*.[10] Again, no further conclusions can be drawn since no ground truth is available.

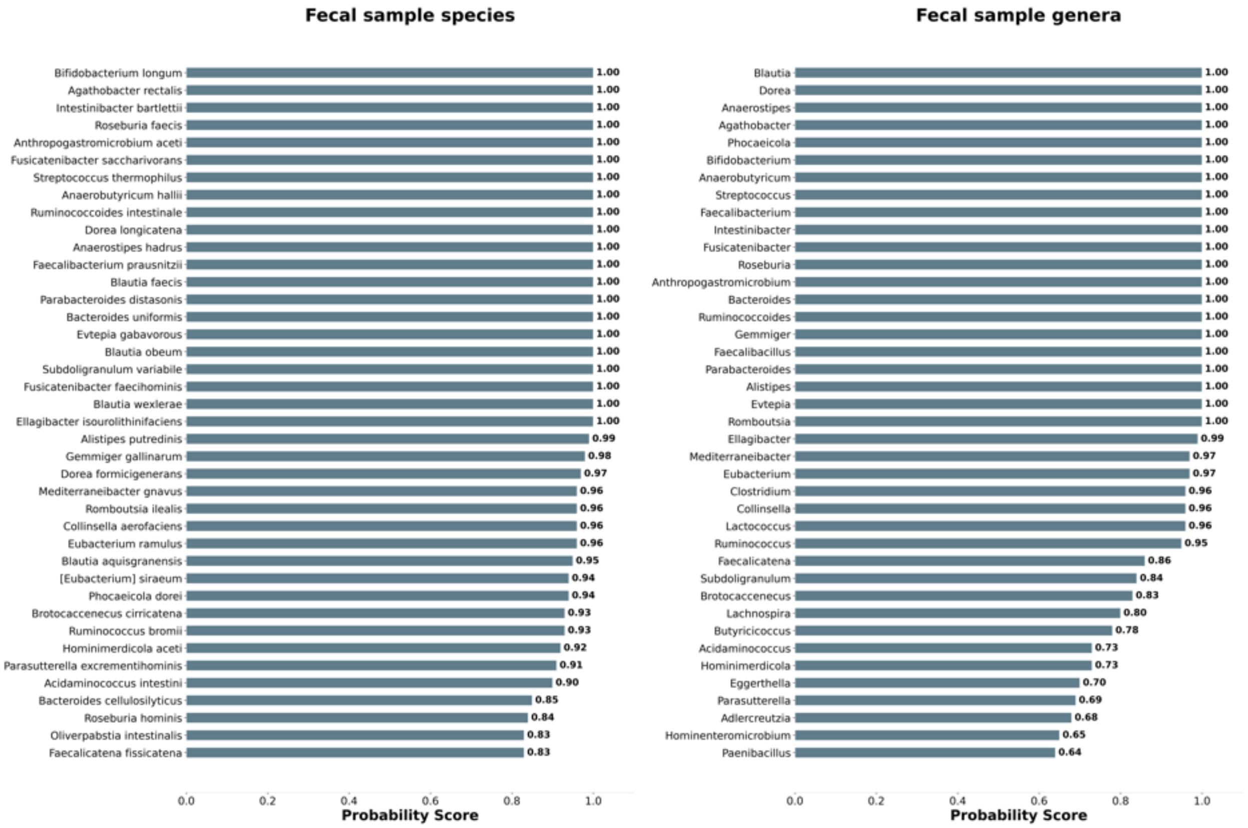

Figure S15: Taxonomic identification of the fecal sample F07 at the genus and species level.

To asses the accuracy of the identification for the ocean sample and the soil sample shown in Figure S16 we compared the identification made by the Peptonizer2000 against the identifications in the original publications (ocean[11] and soil[12]). To help asses accuracy, we compare the Peptonizer's results with the original results in tabular format in tables T6 for soil and T7 for ocean. Even though the Peptonizer does not use a database tailored for microbiomes such as MGnify[13] or GTDB,[14] most phyla or classes are detected. For both, taxa absent from the original results are detected aswell, however, some taxa were originally reported as 'unclassified' or 'no LCA', which might include these.

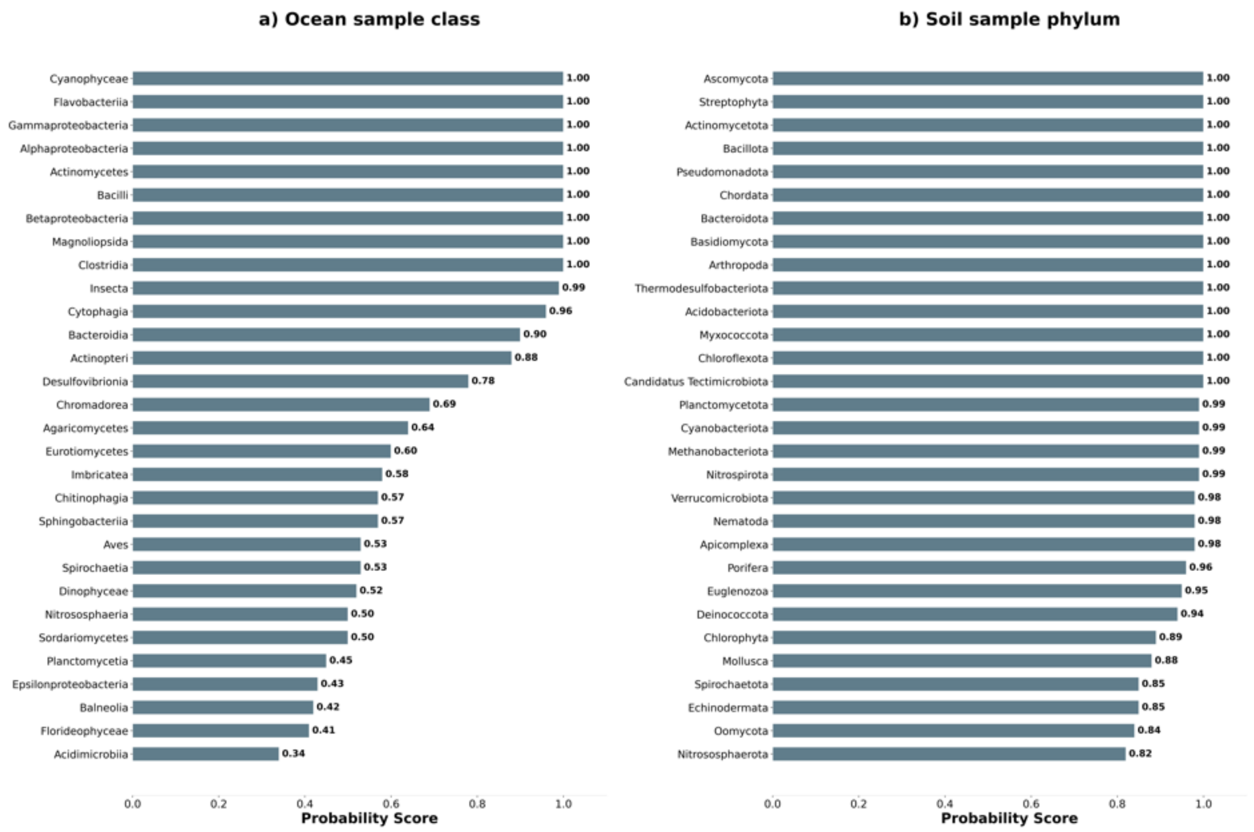

Figure S16: Taxonomic identification of a) the ocean sample at class level and b) the soil sample at phylum level. 30 highest scoring taxa are shown.

Table S6: Taxa detected in the original publication for the soil sample, and whether they were identified by the Peptonizer or not.

**Bacteria Phyla**	**Detected**	**Eukaryota Phyla**	**Detected**
Cyanobacteria	✓	Ciliophora	✓/x (score 0.66)
Gemmatimonadetes	✓/x (score 0.7)	Cnidaria	✓/x (score 0.7)
Proteobacteria (Pseudomonadota)	✓	Mollusca	✓
Nitrospirae	✓	Mucoromycota	✓/x (score 0.64)
Candidatus Rokubacteria	x	Phaeophyceae	x
Verrucomicrobia	✓	Apicomplexa	✓
Planctomycetes	✓	Bacillariophyta	✓
Chloroflexi	✓	Euglenozoa	✓
Firmicutes (Bacillota)	✓	Nematoda	✓
Bacteroidetes	✓	Ascomycota	✓
Acidobacteria	✓	Chlorophyta	✓
Actinobacteria (Actinomycetota)	✓	Arthropoda	✓
		Basidiomycota	✓
		Streptophyta	✓
		Chordata	✓

Table S7: Taxa reported in the original ocean publication and whether their were detected by the Peptonizer or not. We removed taxa such as 'other','no LCA' or 'unclassified' from the original list.

**Taxa**	**Detected**
Cyanobacteria	✓
Alphaproteobacteria	✓
Gammaproteobacteria	✓
Flavobacteria	✓
Proteobacteria	✓
Cytophagia	✓
Actinobacteria (Actinomycetes)	✓
Bacteroidetes	✓
Deltaproteobacteria	x
Saprospiria	x
Planctomycetia	x

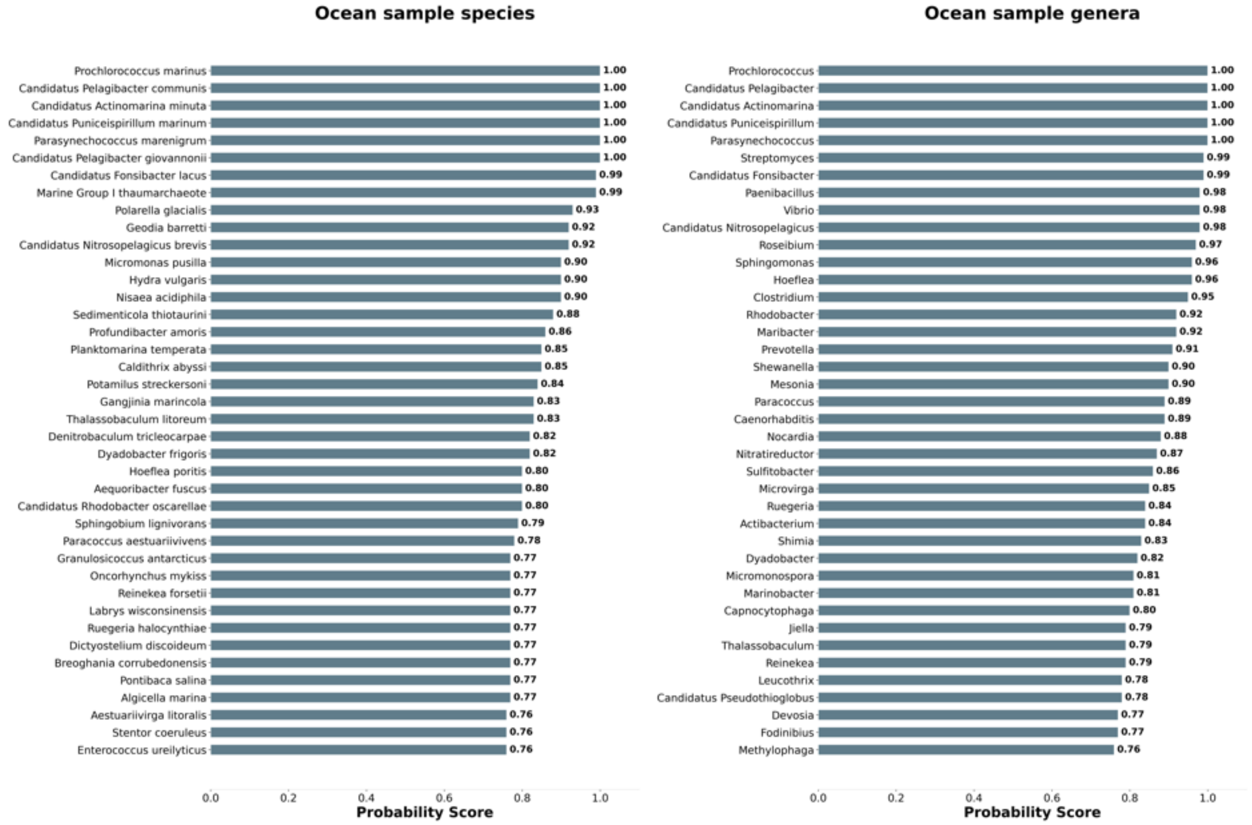

Figure S17: Taxonomic identification of the ocean sample at the species and genus level. 40 highest scoring taxa shown.

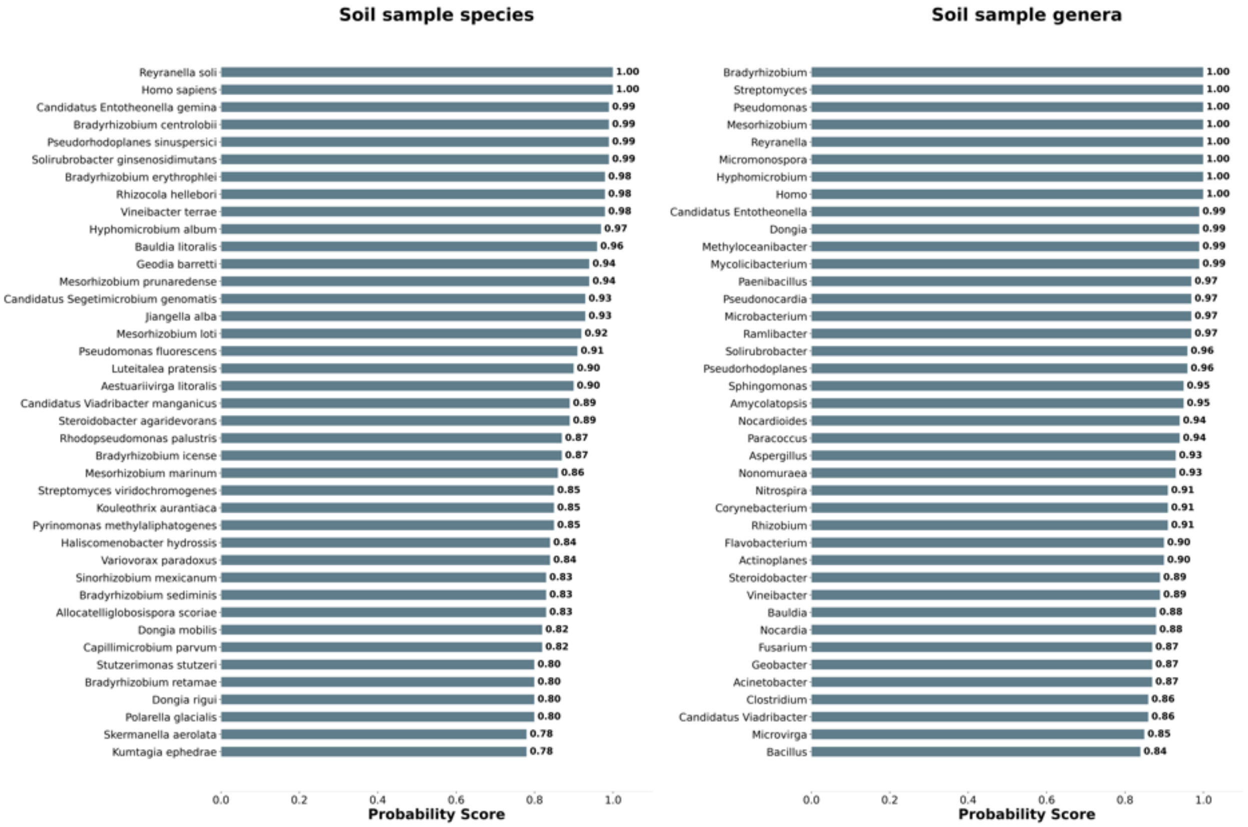

Figure S18: Taxonomic identification of the soil sample at the species and genus level.

References

(1) Huerta-Cepas, J.; Serra, F.; Bork, P. ETE 3: Reconstruction, Analysis, and Visualization of Phylogenomic Data. *Molecular Biology and Evolution* **2016**, *33*, 1635–1638.

(2) McKinney, W.; others Data structures for statistical computing in python. Proceedings of the 9th Python in Science Conference. 2010; pp 51–56.

(3) Hagberg, A. A.; Schult, D. A.; Swart, P. J. Exploring Network Structure, Dynamics, and Function using NetworkX. Proceedings of the 7th Python in Science Conference. Pasadena, CA USA, 2008; pp 11 – 15.

(4) Virtanen, P. et al. scipy/scipy: SciPy 0.19.0. *Zenodo* **2017**, Publisher: Zenodo ADS Bibcode: 2017zndo....376244V.

(5) Cock, P. J. A.; Antao, T.; Chang, J. T.; Chapman, B. A.; Cox, C. J.; Dalke, A.; Friedberg, I.; Hamelryck, T.; Kauff, F.; Wilczynski, B.; de Hoon, M. J. L. Biopython: freely available Python tools for computational molecular biology and bioinformatics. *Bioinformatics* **2009**, *25*, 1422–1423.

(6) Hunter, J. D. Matplotlib: A 2D graphics environment. *Computing in Science & Engineering* **2007**, *9*, 90–95.

(7) Waskom, M. L. seaborn: statistical data visualization. *Journal of Open Source Software* **2021**, *6*, 3021.

(8) Kleiner, M.; Thorson, E.; Sharp, C. E.; Dong, X.; Liu, D.; Li, C.; Strous, M. Assessing species biomass contributions in microbial communities via metaproteomics. *Nature Communications* **2017**, *8*, 1558, Number: 1 Publisher: Nature Publishing Group.

(9) Ramirez Garcia, A.; Greppi, A.; Constancias, F.; Ruscheweyh, H.-J.; Gasser, J.; Hurley, K.; Sturla, S. J.; Schwab, C.; Lacroix, C. Anaerobutyricum hallii promotes the

functional depletion of a food carcinogen in diverse healthy fecal microbiota. *Frontiers in Microbiomes* **2023**, *2*, Publisher: Frontiers.

(10) Zhou, J.; Wu, X.; Li, Z.; Zou, Z.; Dou, S.; Li, G.; Yan, F.; Chen, B.; Li, Y. Alterations in Gut Microbiota Are Correlated With Serum Metabolites in Patients With Insomnia Disorder. *Frontiers in Cellular and Infection Microbiology* **2022**, *12*, Publisher: Frontiers.

(11) Saito, M. A. et al. Results from a multi-laboratory ocean metaproteomic intercomparison: effects of LC-MS acquisition and data analysis procedures. *Biogeosciences* **2024**, *21*, 4889–4908, Publisher: Copernicus GmbH.

(12) Jouffret, V.; Miotello, G.; Culotta, K.; Ayrault, S.; Pible, O.; Armengaud, J. Increasing the power of interpretation for soil metaproteomics data. *Microbiome* **2021**, *9*, 195.

(13) Richardson, L. et al. MGnify: the microbiome sequence data analysis resource in 2023. *Nucleic Acids Research* **2023**, *51*, D753–D759.

(14) Chaumeil, P.-A.; Mussig, A. J.; Hugenholtz, P.; Parks, D. H. GTDB-Tk v2: memory friendly classification with the genome taxonomy database. *Bioinformatics* **2022**, *38*, 5315–5316.
